# Ecological succession and topological rewiring of the oral microbiome in giant panda cubs

**DOI:** 10.1186/s12866-026-05477-8

**Published:** 2026-07-31

**Authors:** Helin Chen, Jiabin Liu, Rong Hou, Fengli Chen, Dunwu Qi, Yu Chen, Wenlei Bi, Yanshan Zhou, Jiang Gu, Guanwei Lan, Wei Wu, Kai Zhang, Rui Ma

**Affiliations:** 1 College of Animal and Veterinary Sciences, Southwest Minzu University, Chengdu, 610000 Sichuan China; 2 Key Laboratory of Animal Medicine of Sichuan Education Department, Southwest Minzu University, Chengdu, 610000 Sichuan China; 3 The Conservation of Endangered Wildlife Key Laboratory of Sichuan Province, Chengdu Research Base of Giant Panda Breeding, Chengdu, 610000 Sichuan China

**Keywords:** *Ailuropoda melanoleuca*, Host ontogeny, Autogenic niche reconstruction, Dental plaque biofilm, Priority effects

## Abstract

In specialized herbivores retaining a carnivorous digestive tract, such as the giant panda, early-life oral microbiome assembly is crucial for lifelong health, yet remains poorly understood. Here, we longitudinally tracked the saliva and supragingival plaque microbiomes of captive cubs (180 matched samples) from birth to one year using microbiome sequencing and topological cartography. We demonstrate a profound spatial-temporal divergence driven by deterministic succession. Initial plaque colonization exhibited transient priority effects reflecting maternal imprints, rapidly superseded by autogenic niche reconstruction and deep anaerobic maturation. Host ontogeny, notably deciduous tooth eruption, triggered intense microbial exchange, causing significant transient habitat homogenization (peak Mantel correlations at months 6 and 8, *p* < 0.001). Mature plaque selectively enriched for peptidoglycan biosynthesis and complex carbohydrate degradation, establishing a pre-adaptive metabolic reservoir for the impending high-fiber bamboo diet. Crucially, topological analysis (interaction edges expanded 3.4-fold) revealed that early pioneers evade competitive exclusion by shifting their ecological niche to become inter-module connectors (Pi > 0.62), providing spatial scaffolds for late-arriving climax communities. These findings decipher the deterministic assembly rules of saliva and plaque microbiomes during the extreme milk-to-bamboo dietary transition, advancing our understanding of giant panda adaptive evolution and identifying native keystone targets for preventive veterinary interventions.

**Supplementary Information:**

The online version contains supplementary material available at https://doi.org/10.1186/s12866-026-05477-8.

## Introduction

The early-life assembly of the host-associated microbiome constitutes a critical developmental window that dictates lifelong metabolic and immunological trajectories [1, 2]. This assembly process reflects an unparalleled ecological imperative in the giant panda (*Ailuropoda melanoleuca*), a species that exemplifies a profound evolutionary paradox: an obligate herbivore enduring a hyper-lignocellulosic bamboo diet while phylogenetically retaining the unspecialized, short gastrointestinal tract of a carnivore [3, 4]. Lacking the complex rumination morphology or extensive hindgut fermentation capacity typical of anatomical herbivores, the giant panda faces extreme physiological constraints during the weaning transition [5]. Consequently, the oral cavity—the primary anatomical gateway for the mechanical disruption and initial enzymatic processing of recalcitrant bamboo foliage—bears an exceptional functional burden [6–8]. Oral health is not merely a clinical metric but a critical determinant of lifelong foraging efficiency and evolutionary fitness in this species [9]. Therefore, decoding the developmental dynamics of the oral microenvironment during the highly vulnerable pre-weaning stage transcends conservation biology; it offers a unique paradigm for understanding how host-microbiome co-evolution acts as a compensatory mechanism for macroscopic anatomical deficits [10].

The mammalian oral microbiome is not a homogenous, planktonic suspension but rather a highly compartmentalized ecosystem, primarily functioning as structured, surface-attached biofilms (i.e., dental plaque) bathed in a continuous flow of saliva [11, 12]. The assembly of these biofilms follows a predictable, orderly ecological succession [13, 14]. It is initiated by “pioneer” species—early colonizers capable of adhering to tooth pellicles and surviving in relatively oxygen-rich environments [15, 16]. Through their metabolic activities, these pioneers progressively modify the local microhabitat, depleting oxygen and creating complex attachment sites that facilitate the colonization of late-arriving “climax” species [17, 18]. This autogenic succession ultimately yields a mature, stable, and highly synergistic climax community equipped with specialized metabolic factories [19, 20]. While this ecological handover from pioneers to climax taxa has been extensively documented in human and murine models, revealing its crucial role in orchestrating local immune maturation and disease resistance, the assembly rules governing the oral biofilm in specialized herbivores like giant pandas remain completely elusive [21, 22].

Despite a recent surge in microbiome research aimed at giant panda conservation, the overwhelming majority of studies have exclusively focused on the gut microbiome of adult individuals, typically utilizing cross-sectional designs to investigate bamboo adaptation [23–25]. The oral microbiome, particularly during the critical first year of life, has been largely relegated to a scientific black box [26]. Furthermore, the few existing oral studies have heavily relied on easily accessible saliva samples, fundamentally failing to distinguish between the transient, planktonic microbes in saliva and the functionally critical, resident biofilm communities in dental plaque [9, 27]. Consequently, we lack a fundamental understanding of how the panda dental plaque is assembled “from scratch”. We do not know which taxa act as the initial pioneers, which establish the climax community, or how these microbial networks undergo topological reorganization to adapt to the impending, rigorous bamboo diet [28]. Capturing this high-resolution, longitudinal trajectory transcends traditional conservation efforts; without identifying the native keystone taxa that orchestrate this microbiome-mediated compensation, we cannot fully decode the adaptive blueprint that permits the giant panda’s extreme dietary specialization, nor can we design evolutionarily informed interventions to safeguard vulnerable captive populations [24, 29].

To bridge this critical knowledge gap, we conducted continuous longitudinal monitoring of oral microbiome assembly in giant panda cubs from birth to one year of age. By leveraging high-throughput microbiome sequencing across 180 precisely matched saliva and supragingival plaque samples, we aimed to test the following hypotheses: (1) dental plaque undergoes profound spatial-temporal divergence from saliva, characterized by autogenic niche reconstruction and a pre-adaptive metabolic shift toward complex carbohydrate degradation; (2) plaque maturation is driven by a deterministic ecological handover, wherein early pioneer taxa evade competitive exclusion by structurally transitioning into inter-module network connectors; and (3) this topological rewiring establishes the fundamental physical scaffold required to maintain community resilience against the intense biomechanical and metabolic stresses of the impending bamboo diet. By deciphering the deterministic assembly rules of this highly specialized oral microenvironment, this investigation aims to provide unprecedented insights into how host-microbiome co-evolution compensates for macroscopic anatomical deficits, thereby offering crucial theoretical foundations for evolutionary ecology and native keystone targets for conservation medicine.

## Materials and methods

### Study cohort and animal husbandry

This longitudinal study was conducted at the Chengdu Research Base of Giant Panda Breeding (Chengdu, China). The cohort consisted of 12 captive giant panda cubs (6 males, 6 females) born via natural labor in 2021. All giant panda cubs included in this study were legally sourced from, bred, and permanently housed at the Chengdu Research Base of Giant Panda Breeding (Chengdu, China). All cubs were maternal-reared with standardized assistance from designated keepers. The dietary protocol was strictly controlled: from birth to 12 months of age, cubs were exclusively fed maternal milk, supplemented with specialized formula during periods of insufficient maternal lactation. None of the enrolled cubs consumed bamboo or bamboo shoots during the entire study period. Cubs were initially housed indoors and gradually transitioned to indoor enclosures equipped with wooden platforms and outdoor playgrounds with natural vegetation. The study protocol was approved by the Institutional Animal Care and Use Committee of the Chengdu Research Base of Giant Panda Breeding (No. 201806).

### Longitudinal sampling and clinical assessment

A total of 180 oral microbiome samples were longitudinally collected from the 12 cubs. Specifically, 108 saliva samples were collected at 1, 2, 3, 4, 5, 6, 8, 10, and 12 months of age. Concurrently, 72 supragingival plaque samples were collected from the same individuals at 4, 5, 6, 8, 10, and 12 months of age (sampling commenced at 4 months corresponding with tooth eruption). This shift in sampling frequency from monthly (months 1–6) to bimonthly (months 8–12) was designed to balance the necessity of capturing rapid ecological dynamics during early critical development with strict animal welfare considerations, minimizing handling stress as the cubs grew larger and more active. Sampling was performed using sterile swabs (Hi-Swab, Tiangen, Beijing, China). Saliva was collected by vigorously swabbing the hard palate, tongue, and buccal mucosa, whereas plaque was collected by swabbing the lingual and occlusal surfaces of all erupted teeth. All samples were immediately snap-frozen on dry ice and stored at -80 °C until further processing. Oral health status was assessed monthly by veterinarians using a standardized dental chart; no clinical diseases or active caries were recorded for any individual during the study period.

### DNA extraction and 16 S rRNA gene sequencing

Genomic DNA was extracted using the TIANamp Swab DNA Kit (Tiangen, Beijing, China) according to the manufacturer’s instructions. DNA quality and concentration were verified by 1.5% agarose gel electrophoresis and a NanoDrop 2000 spectrophotometer, respectively. The V3–V4 hypervariable regions of the bacterial 16 S rRNA gene were amplified using universal primers 338 F (5′-ACTCCTACGGGAGGCAGCAG-3′) and 806R (5′-GGACTACGCGGGTATCTAAT-3′). High-throughput sequencing was performed on the Illumina NovaSeq 6000 platform to generate 2 × 250 bp paired-end reads at Novogene Bioinformatics Technology Co., Ltd. (Tianjin, China).

### Bioinformatics processing and community diversity metrics

Raw sequencing data were demultiplexed and quality-filtered using QIIME 2 (v2022.2) [30]. High-quality sequences were clustered into Operational Taxonomic Units (OTUs) at 97% sequence identity using UPARSE (v11.0), followed by chimera removal via UCHIME [31]. This 97% clustering threshold was deliberately selected to mitigate the potential artificial over-splitting of taxa caused by intragenomic 16 S rRNA sequence heterogeneity, thereby ensuring conservative diversity estimations and robust topological inferences for the downstream co-occurrence network analyses. Taxonomy was assigned against the SILVA 128 database using a naive Bayes classifier (confidence threshold: 70%) [32]. This database version was employed as part of our standardized bioinformatic pipeline at the time of the unified data analysis. Importantly, while taxonomic nomenclatures have recently evolved (e.g., SILVA 138 or GTDB), our core macro-ecological inferences (including diversity metrics, source-tracking, and network topologies) are mathematically derived from sequence-based abundance matrices and remain fundamentally independent of these taxonomic reclassifications. Non-bacterial sequences (chloroplasts and mitochondria) were strictly filtered out. Prior to diversity calculations, the OTU abundance table was rarefied to the minimum sequencing depth of 30,333 reads per sample across all samples to ensure unbiased comparisons.

All subsequent statistical analyses and visualizations were performed using Python (v3.10.12) and GraphPad Prism 9 (GraphPad Software, CA, USA). Alpha diversity was quantified using the Chao1 richness estimator and the Shannon index. Beta diversity was evaluated based on Bray-Curtis dissimilarity matrices calculated via the scipy.spatial.distance module (v1.11.2) and visualized using Principal Coordinate Analysis (PCoA). To partition the variance in community structure explained by spatial niche and host identity, Permutational Multivariate Analysis of Variance (PERMANOVA) was executed with 999 permutations. To stringently control for the longitudinal and repeated-measures design, the PERMANOVA permutations were strictly constrained within each individual host (Panda_ID block) to properly account for sample non-independence and individual-specific baselines.

To evaluate longitudinal trends while accounting for repeated measures, Linear Mixed-Effects Models (LMM) were constructed using the statsmodels package (v0.14.0) [33]. Developmental time (Month) and spatial habitat (Niche) were designated as fixed effects. Crucially, the individual host identity (Panda_ID) was assigned as a random effect to rigorously control for individual-specific baseline variations and dynamic confounding factors throughout the developmental stages, specifically the fluctuating formula-to-maternal milk supplementation ratios and varying frequencies of maternal contact. P-values derived from LMMs were adjusted for multiple comparisons using the Benjamini-Hochberg False Discovery Rate (FDR) procedure, with statistical significance defined as *p*_FDR_ < 0.05.

### Dynamic microbial source tracking and structural correlations

To evaluate the chronological assembly of the dental plaque microbiome, a dynamic microbial source tracking approach based on a Non-Negative Least Squares (NNLS) optimization algorithm was developed via the scipy.optimize.nnls module [34]. For each sampled plaque community (sink) at a specific developmental month (M), three potential source reservoirs were constructed: (1) historical pre-eruption saliva pool (aggregated host saliva from months 1–3); (2) concurrent own saliva at month M; and (3) population background saliva at month M. While the latter two concurrent reservoirs inevitably exhibit partial collinearity due to shared dietary, environmental, and seasonal drivers, they were modeled separately to conceptually distinguish individual-specific localized cross-talk from general cohort-level environmental exposure. The NNLS framework intrinsically accommodates this by constraining coefficients to non-negative values, thereby preventing erratic variance inflation and ensuring a robust competitive assignment. The NNLS algorithm computed non-negative coefficients, which were converted to relative percentage contributions, with the remaining fraction classified as plaque-specific unknowns. Longitudinal shifts in these contributions were statistically evaluated using LMMs. Furthermore, structural correlations reflecting historical and concurrent ecological dynamics were assessed using Mantel tests based on Spearman rank correlations with 9999 random matrix permutations.

### Taxonomic biomarker discovery and longitudinal taxa tracking

To identify stage-specific differential biomarkers, Linear discriminant analysis Effect Size (LEfSe) was performed via the Galaxy online platform [35]. The alpha value for the factorial Kruskal-Wallis test was set to 0.05, and the threshold on the logarithmic LDA score was strictly set to > 3.0. For saliva, comparisons were made across three stages (months 1–3, 4–6, and 8–12); for dental plaque, comparisons were conducted between early (months 4–6) and mature (months 8–12) stages.

To precisely define and trace the ecological roles of specific OTUs during plaque assembly, a rigorous mathematical screening approach was developed [36]. These specific relative abundance thresholds (e.g., > 1.0%) were strategically implemented based on established microbial ecology conventions to filter out transient environmental members and stochastic sequencing noise. This approach ensured that our screening focused strictly on the dominant, functionally active core microbiome, while simultaneously reducing the multiple-testing burden for downstream statistical validations. Consequently, “Pioneer” taxa were defined by three concurrent conditions: (1) mean relative abundance > 1.0% in pre-eruption saliva; (2) abundance > 1.0% in early plaque (month 4); and (3) plaque abundance at month 4 must exceed 1.5 times the abundance at month 12. Conversely, “Climax” taxa were defined by: (1) abundance < 0.5% in pre-eruption saliva; (2) abundance > 1.0% in mature plaque (month 12); and (3) plaque abundance at month 12 must exceed 2.0 times the abundance at month 4. Temporal dynamics of these mathematically screened OTUs were statistically validated using LMMs (*p*_FDR_ < 0.05).

### Functional prediction and co-occurrence network analysis

Metabolic pathways were predicted from 16 S rRNA sequences using PICRUSt2 (v2.3.0) [37]. Pathways with a mean relative abundance < 0.05% were filtered out. Biofilm-specific metabolic biomarkers driven by plaque maturation were identified using LMMs, and their co-occurrence patterns with the 7 keystone OTUs (pioneers and climax taxa) were evaluated utilizing FDR-corrected Spearman’s rank correlations.

To investigate the topological evolution of species interactions, microecological co-occurrence networks were constructed for early (4–6 months) and mature (8–12 months) plaque. Given the non-normal distribution of microbiome data, we employed the non-parametric Spearman rank correlation method, which assumes monotonic rather than strictly linear relationships. The pairwise correlation matrices were calculated using the scipy.stats and networkx packages in Python. To ensure robust statistical support and avoid spurious correlations, each temporal network was constructed utilizing a large sample size of 36 independent samples (12 individuals × 3 developmental months). Dominant OTUs (> 0.05% mean relative abundance) were retained, and edges were strictly filtered to include only robust interactions (|r| > 0.6, *p* < 0.01). Network visualization and topological parameter extraction were conducted in Gephi (v0.10.1) applying the Fruchterman-Reingold layout.

To quantify topological importance and identify keystone species, within-module connectivity (Zi) and among-module connectivity (Pi) were calculated based on the Louvain modularity algorithm. Nodes were subsequently classified into peripherals (Zi ≤ 2.5, Pi ≤ 0.62), connectors (Zi ≤ 2.5, Pi > 0.62), module hubs (Zi > 2.5, Pi ≤ 0.62), and network hubs (Zi > 2.5, Pi > 0.62) according to classical ecological network criteria, an approach proven highly effective in evaluating microbiome network resilience [38, 39].

## Results

### High ecological concordance and host-individualized succession characterize the early developing oral microbiome

**Fig. 1:**
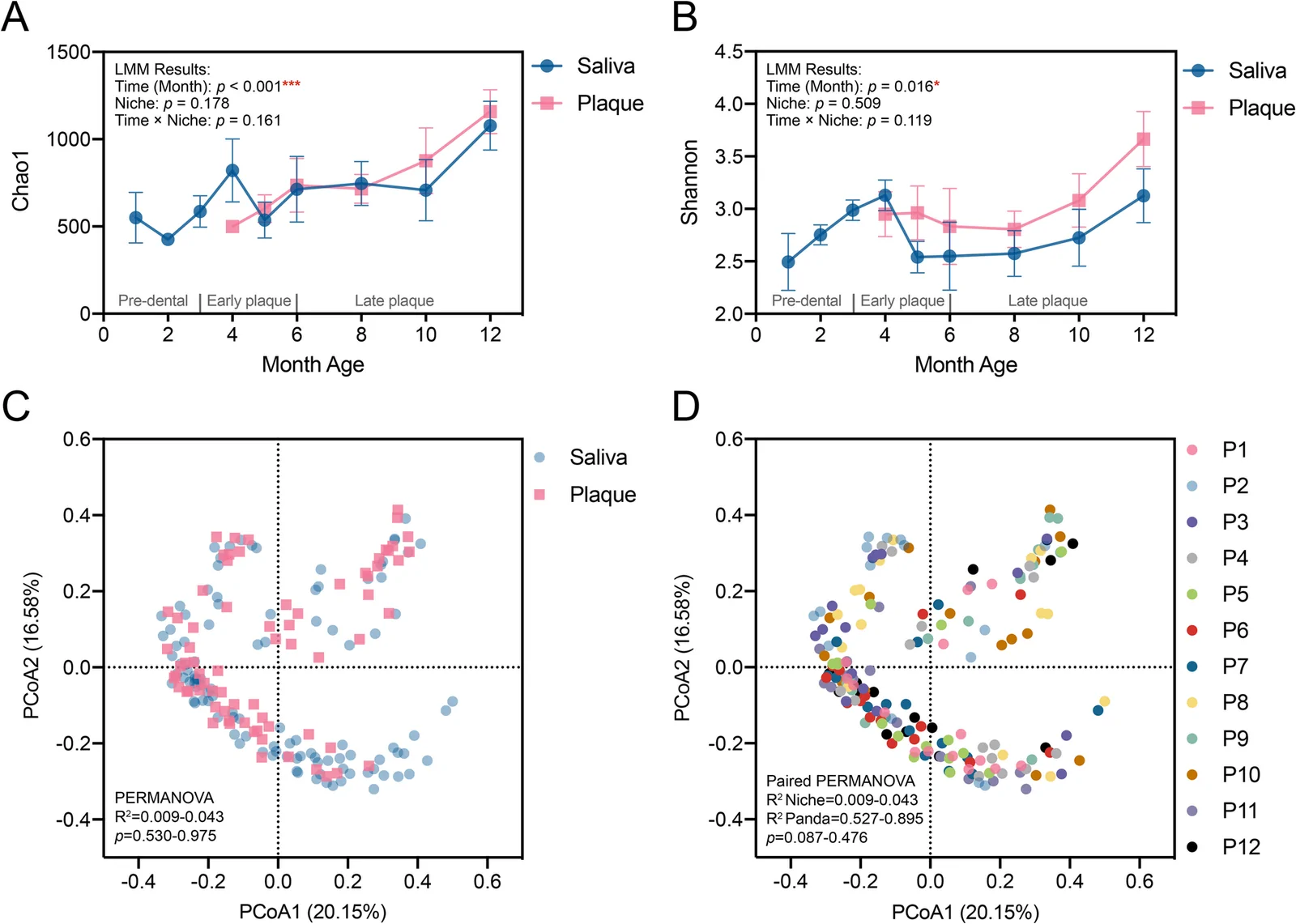
Longitudinal developmental dynamics and structural variance of the early oral microbiome in giant panda cubs. Alpha diversity of the oral microbial communities evaluated by the (**A**) Chao1 richness estimator and the (**B**) Shannon diversity index over a 12-month period. Solid lines represent the mean values for the saliva (blue circles) and dental plaque (red squares) communities at each sampled month. Error bars indicate SEM, and significance was determined by LMM. (* *p* < 0.05, ** *p* < 0.01, *** *p* < 0.001) (**C**) Principal Coordinate Analysis (PCoA) plot based on Bray-Curtis dissimilarity, colored by spatial niche. (**D**) The identical PCoA ordination space colored by host identity (Panda_ID) to illustrate individual-specific trajectories

To investigate the developmental dynamics and spatial differentiation of the oral microbiome in giant panda cubs during the first year of life, we longitudinally profiled the microbial communities of 180 samples from two distinct niches (saliva and dental plaque) across 12 individuals. Alpha diversity was evaluated using the Chao1 richness estimator (Fig. 1A) and the Shannon diversity index (Fig. 1B). Linear mixed-effects models (LMM), revealed a significant continuous increase in community richness over time (Month effect for Chao1: Estimate = 71.56, Z = 3.859, *p* = 0.0001, Table S1, Table S2). Similarly, the overall community diversity and evenness significantly expanded as the cubs developed (Month effect for Shannon: Estimate = 0.076, Z = 2.388, *p* = 0.016, Table S1, Table S2). Notably, neither the Chao1 nor the Shannon index exhibited significant differences between the planktonic saliva and the biofilm plaque niches (Niche effect for Chao1: *p* = 0.178; Shannon: *p* = 0.509, Fig. 1A, Table S1, S2). Furthermore, the interaction term between time and niche was not statistically significant (Chao1: *p* = 0.161; Shannon: *p* = 0.119), indicating a synchronized and parallel developmental trajectory of the microbial communities across both habitats (Fig. 1B, Table S1, S2).

We subsequently assessed beta diversity using Principal Coordinate Analysis (PCoA) based on Bray-Curtis distance matrices to visualize structural variations. When samples were grouped by spatial niches, saliva and plaque communities exhibited extensive structural overlap along the first two principal coordinates, which explained 20.15% and 16.58% of the total variance, respectively (Fig. 1C). Permutational multivariate analysis of variance (PERMANOVA) across respective developmental months confirmed that the spatial niche exerted a minimal and non-significant effect on the overall community composition (R^2^ ranging from 0.009 to 0.043, all *p* > 0.530, Fig. 1C, Table S3). Conversely, when the same ordination space was colored by host identity, strong structural clustering was observed (Fig. 1D). Paired PERMANOVA demonstrated that inter-individual variation was the primary driver of the oral microbiome structure, explaining a substantial proportion of the total variance across different developmental stages (Individuals R^2^ ranging from 0.503 to 0.895, Fig. 1D, Table S3). Overall, the physical eruption of teeth was not associated with a statistically detectable immediate spatial divergence of the oral microbiota. Instead, the early oral cavity functioned as a highly integrated ecological system, governed predominantly by individual-specific temporal succession rather than spatial niche segregation.

### Dynamic assembly modeling reveals a shift from historical legacy to concurrent cross-talk in plaque microbiome

**Fig. 2:**
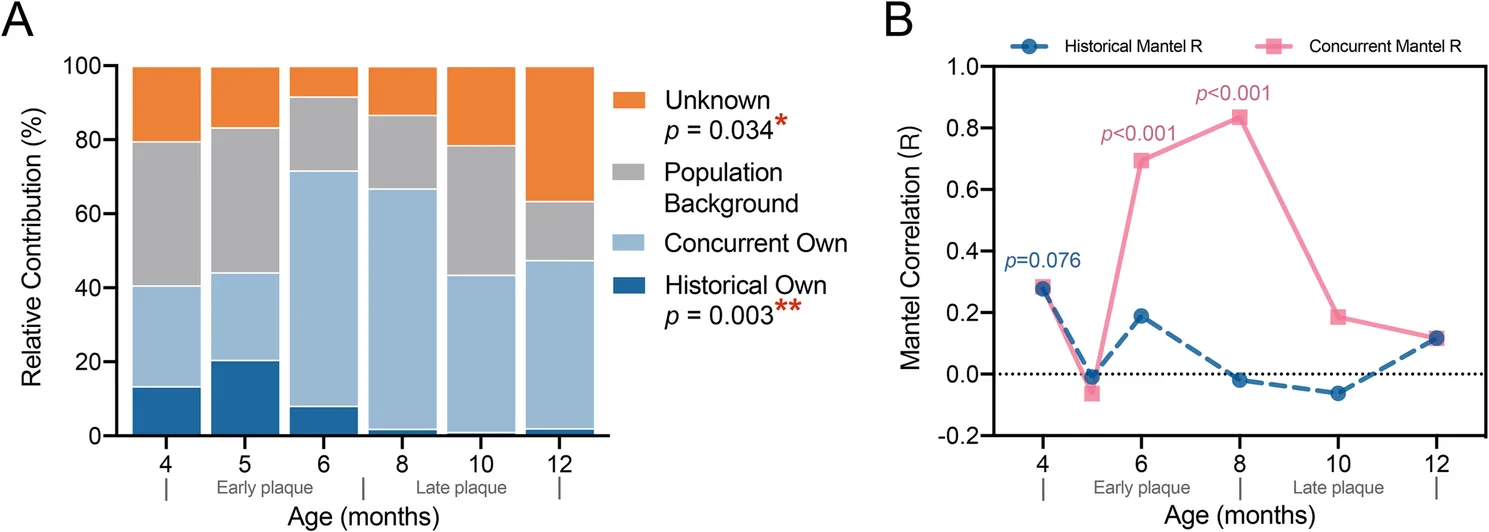
Dynamic assembly and structural correlations of the developing dental plaque microbiome. (**A**) Stacked bar chart showing the fractional contributions of four microbial source reservoirs to the dental plaque community from month 4 to month 12, based on dynamic NNLS estimation. Asterisks indicate significant temporal changes (* *p* < 0.05, ** *p* < 0.01). (**B**) Dynamic shifts in structural correlations (Mantel R statistic) between salivary reservoirs and plaque communities over time. The blue dashed line traces the historical correlation (months 1–3 saliva vs. plaque), while the solid red line traces the concurrent correlation (saliva vs. plaque matched at the exact developmental month). *P*-values are annotated for significant or marginally significant data points

Dynamic microbial source tracking (Fig. 2A) revealed that at month 4, the nascent plaque community was characterized by a historical legacy, with the host’s own pre-eruption saliva pool contributing a mean of 13.45% to the total community (Fig. 2A, Table S4). Linear mixed-effects models (LMM) incorporating host identity as a random effect demonstrated that this historical legacy underwent a highly significant temporal decay (Estimate = -2.057, *p* = 0.003), diminishing to 2.11% by month 12 (Fig. 2A, Table S4, S5). During the subsequent developmental stages, the plaque assembly was heavily dominated by real-time microbial exchange with the host’s concurrent saliva, which contributed 63.59% at month 6 and peaked at 64.92% at month 8 (temporal trend not significant, *p* = 0.178, Fig. 2A, Table S4, S5). Concurrently, the unassigned fraction of the community, representing plaque-specific specialized taxa, exhibited a statistically significant continuous expansion over time (Estimate = 2.091, *p* = 0.034), ultimately accounting for 36.53% of the mature biofilm at month 12 (Fig. 2A, Table S4, S5).

To structurally validate the transition from an early historical legacy to concurrent ecological coupling, dynamic Mantel tests were conducted using Spearman rank correlations (Fig. 2B). The structural correlation between the historical pre-eruption saliva matrix and the plaque matrix reached its maximum at month 4 with marginal significance (*R* = 0.277, *p* = 0.076) and subsequently declined to non-significant levels (Fig. 2B, Table S6). In contrast, the concurrent structural correlation between the saliva and plaque matrices of the same developmental month became highly significant during the intermediate developmental window, specifically at month 6 (*R* = 0.694, *p* < 0.001) and month 8 (*R* = 0.835, *p* < 0.001, Fig. 2B, Table S6). In conclusion, the initial colonization of dental plaque retains a marginally significant historical legacy from the pre-eruption salivary microbiome. This potential early historical legacy is rapidly superseded by intense concurrent microbial cross-talk with the flowing saliva and the continuous, significant maturation of a spatially specialized biofilm community.

### Taxonomic succession and identification of stage-specific pioneer and climax taxa during biofilm maturation

**Fig. 3:**
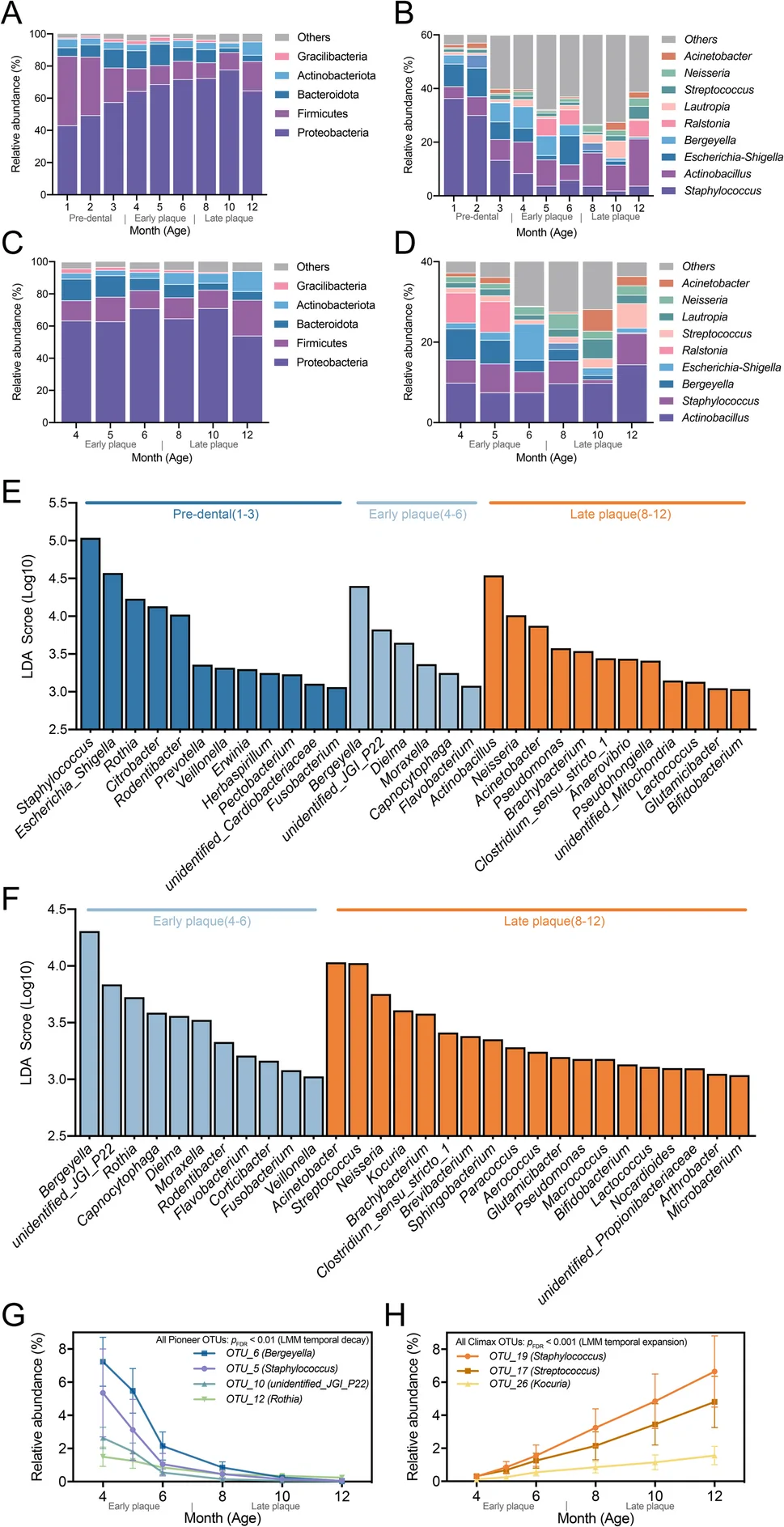
Taxonomic composition, stage-specific biomarkers, and longitudinal trajectories of keystone pioneer and climax taxa. Relative abundance of the top 5 phyla in (**A**) saliva and (**C**) dental plaque, and the top 9 genera in (**B**) saliva and (**D**) plaque over the developmental timeline. Stage-specific taxonomic biomarkers identified by LEfSe (LDA > 3.0, *p* < 0.05) in (**E**) saliva and (**F**) plaque ecosystems. Longitudinal relative abundances (Mean ± SEM) of the identified (**G**) pioneer OTUs and (H) climax OTUs in dental plaque over time. Temporal trends were evaluated by LMM

To characterize the temporal shifts in microbial community composition, taxonomic profiles were evaluated at the phylum and genus levels. Throughout the 12-month developmental period, the salivary microbiome was consistently dominated by Proteobacteria, Firmicutes, and Bacteroidota at the phylum level (Fig. 3A, Table S7), with *Actinobacillus*, *Streptococcus*, and *Neisseria* representing the predominant genera (Fig. 3B; Table S8). In contrast, the nascent dental plaque at month 4 exhibited a distinct compositional profile heavily dominated by Proteobacteria (Mean = 63.43%, SD = 14.28%), followed by Bacteroidota (13.38%) and Firmicutes (12.57%, Fig. 3C, Table S7). As the plaque matured from month 4 to month 12, a progressive taxonomic succession was observed at the genus level (Fig. 3D; Table S8), characterized by the gradual decline of early colonizers such as *Bergeyella* and the subsequent enrichment of biofilm-associated genera, including *Streptococcus* and *Kocuria*. This stage-specific structural divergence across pre-eruption saliva (months 1–3), early plaque (months 4–6), and mature plaque (months 8–12) was further visually and statistically confirmed by principal coordinate analysis coupled with global and pairwise PERMANOVA tests (Figure S1).

To identify statistically significant microbial biomarkers associated with these temporal successions, Linear discriminant analysis Effect Size (LEfSe) was performed. Within the salivary reservoir, three distinct developmental stages were compared (months 1–3, 4–6, and 8–12). The pre-eruption saliva (months 1–3) was significantly enriched with taxa including *Staphylococcus* (LDA = 5.05, *p* < 0.001) and *Rothia* (LDA = 4.22, *p* < 0.001, Figure E, Table S9). The transitional phase (months 4–6) was marked by the enrichment of *Bergeyella* (LDA = 4.40, *p* < 0.001), while the late-stage saliva (months 8–12) showed significant enrichment of *Actinobacillus* (LDA = 4.55, *p* = 0.005) and *Neisseria* (LDA = 3.96, *p* < 0.001, Fig. 3E, Table S9). Concurrently, LEfSe analysis of the dental plaque communities revealed stage-specific structural segregation. The early plaque (months 4–6) was significantly characterized by *Bergeyella* (LDA = 4.30, *p* < 0.001) and *Rothia* (LDA = 3.72, *p* = 0.002, Figure F, Table S9). Conversely, the mature plaque (months 8–12) was distinguished by the significant accumulation of *Acinetobacter* (LDA = 4.03, *p* = 0.045), *Streptococcus* (LDA = 4.02, *p* = 0.018), and *Kocuria* (LDA = 3.60, *p* = 0.002, Fig. 3F, Table S9).

A further strict mathematical screening and LMM test were applied to track the longitudinal trajectories of specific OTUs at the plaque surface to elucidating the strain-level niche specialization characterizing this ecological turnover. Four OTUs were identified as keystone pioneer taxa, which exhibited high initial abundances followed by significant temporal decay. Notably, *Staphylococcus* OTU_5 (Estimate = -0.880, *p*_FDR_ < 0.001) and *Bergeyella* OTU_6 (Estimate = -0.829, *p*_FDR_ < 0.001) were nearly eliminated from the mature plaque community (Fig. 3G; Table S10, S11). Conversely, three OTUs were identified as keystone climax taxa, demonstrating exponential accumulation during biofilm maturation. *Streptococcus* OTU_17 (Estimate = 0.484, *p*_FDR_ < 0.001) and *Staphylococcus* OTU_19 (Estimate = 0.653, *p*_FDR_ < 0.001) exhibited highly significant temporal expansion, establishing dominance in the mature plaque (Fig. 3H; Table Table S10, S11). In summary, the maturation of the dental plaque microbiome is characterized by a consistent directional succession, transitioning from an early community colonized by transient salivary pioneers (e.g., *Bergeyella*) to a highly specialized, mature biofilm dominated by climax taxa (e.g., *Streptococcus*).

### Ecological handover is associated with the biofilm-specific functional maturation of dental plaque

**Fig. 4:**
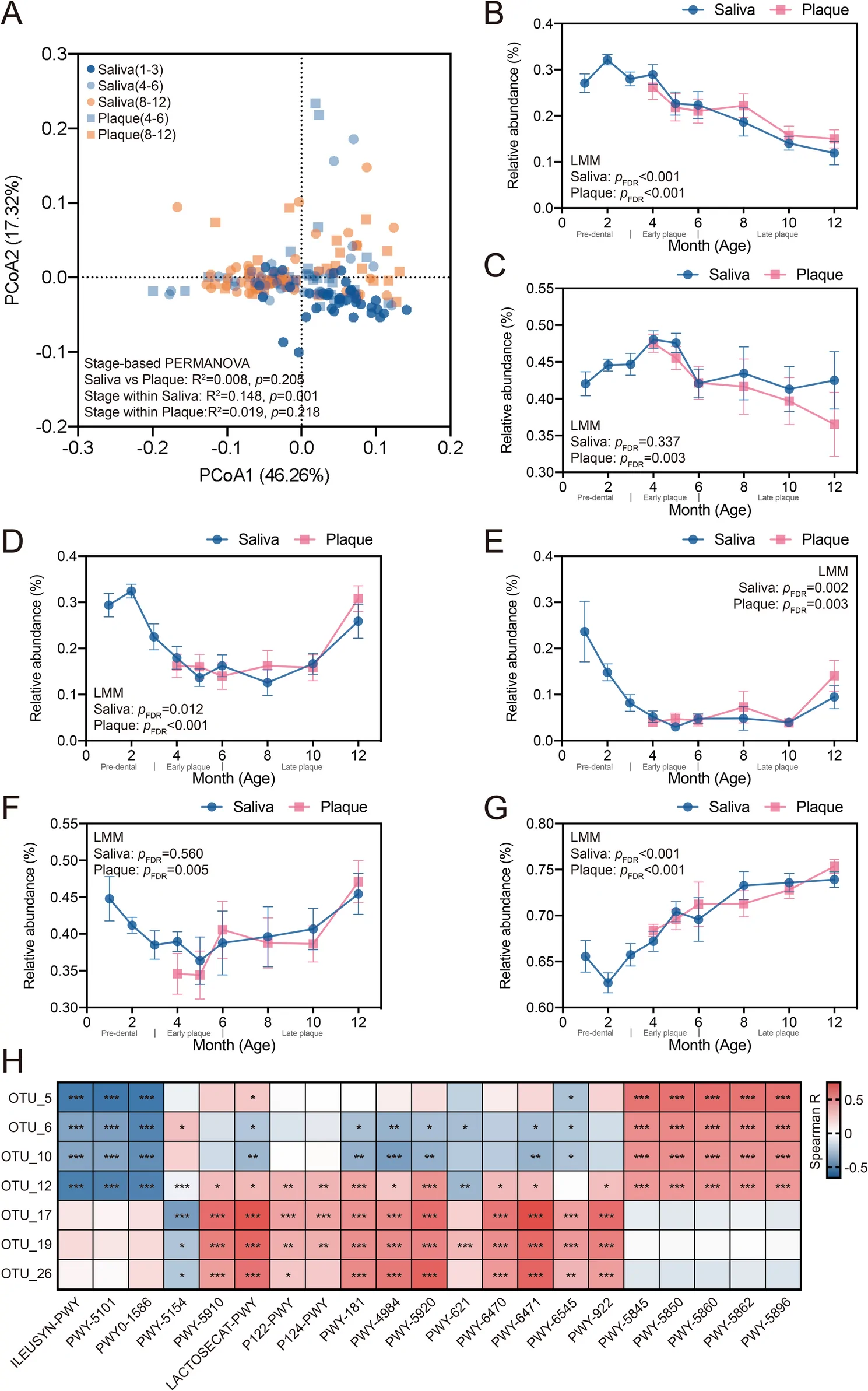
Functional succession of the oral microbiome and its co-occurrence with keystone taxa during early developmental stages. (**A**) Principal Coordinates Analysis (PCoA) plot of PICRUSt2-predicted functional pathways, colored by developmental stage. (**B**-**G**) Longitudinal dynamics of representative metabolic pathways identified by Linear Mixed-Effects Models (LMM). Trajectories of pioneer pathways, including (**B**) PWY-5896 and (**C**) PWY-5154, alongside climax pathways, including (**D**) PWY-6471, (**E**) LACTOSECAT-PWY, (F) PWY-621, and (**G**) ILEUSYN-PWY, are shown. Error bars indicate SEM. (**H**) Spearman correlation heatmap illustrating the co-occurrence patterns between 21 significantly shifted metabolic pathways and 7 keystone OTUs in dental plaque. Asterisks denote statistical significance (* *p*_FDR_ < 0.05, ** *p*_FDR_ < 0.01, *** *p*_FDR_ < 0.001)

While the taxonomic composition of both saliva and plaque microbiomes underwent significant shifts during early development, their functional trajectories exhibited fundamentally divergent patterns (Fig. 4A). To assure the reliability of these 16 S-based functional inferences, the Nearest Sequenced Taxon Index (NSTI) was evaluated. The mean NSTI across all 180 samples was 0.0655 ± 0.0205 (Table S19), indicating a high phylogenetic proximity to reference genomes and robust prediction accuracy for this mammalian oral cohort. Principal Coordinates Analysis (PCoA) revealed that the salivary microbiome remained highly dispersed and fluctuated continuously across the 1–12 month period (PERMANOVA, *p* = 0.001, Fig. 4A, Table S12). Conversely, the dental plaque microbiome rapidly converged into a tightly clustered, highly stable functional climax state by 8–12 months (*p* = 0.218), indicating a highly convergent functional stabilization associated with biofilm assembly (Fig. 4A, Table S12). To delineate the specific metabolic adaptations underpinning this maturation, Linear Mixed-Effects Models (LMM) were utilized to track pathway dynamics, identifying a core set of 21 metabolic pathways undergoing significant succession (*p*_FDR_ < 0.05) exclusively or dominantly in dental plaque. Notably, the early phase of plaque development was marked by the global decline of aerobic and basic metabolic traits. Specifically, aerobic respiration driven by Menaquinol-10 biosynthesis (PWY-5896) exhibited a precipitous and highly significant decline in both saliva (Slope = -0.018, *p*_FDR_ < 0.001) and plaque (Slope = -0.013, *p*_FDR_ = 0.001) niches (Fig. 4B, Table S13, S14), marking a macroscopic transition towards a more anaerobic oral microenvironment. Similarly, early-stage basic metabolism, such as L-arginine biosynthesis (PWY-5154), decreased significantly in dental plaque (Slope = -0.012, *p*_FDR_ = 0.003) while remaining relatively unchanged in saliva (*p*_FDR_ = 0.337, Fig. 4C, Table S13, S14), suggesting a progressive functional decline of aerobic metabolic traits within the biofilm.

Intriguingly, the late-stage plaque maturation was predominantly characterized by the biofilm-specific enrichment of structural and dietary-adaptive pathways. Peptidoglycan biosynthesis (PWY-6471), essential for Gram-positive cell wall construction, increased significantly only in plaque (Slope = 0.014, *p*_FDR_ = 0.001) while significantly declining in saliva (Slope = -0.008, *p*_FDR_ = 0.012, Fig. 4D, Table S13, S14), physically echoing the taxonomic transition towards a Firmicutes-dominated biofilm. Furthermore, during the late developmental stages (months 8–12), distinct plaque-specific functional profiles were established. Specifically, Pathways for lactose and galactose degradation (LACTOSECAT-PWY) surged in the plaque biofilm (Slope = 0.009, *p*_FDR_ = 0.003) despite a concurrent decline in saliva (Slope = -0.010, *p*_FDR_ = 0.002, Fig. 4E, Table S13, S14). Sucrose degradation (PWY-621) also exclusively enriched in plaque (Slope = 0.013, *p*_FDR_ = 0.005) with no significant fluctuation in saliva (*p*_FDR_ = 0.560, Fig. 4F, Table S13, S14). The concurrent upregulation of branched-chain amino acid synthesis, such as L-isoleucine (ILEUSYN-PWY; Plaque Slope = 0.008, *p*_FDR_ = 0.001), further suggested potential metabolic adaptations of the mature plaque (Fig. 4G, Table S13, S14).

To investigate the co-occurrence patterns between the taxonomic succession and these functional shifts, we performed a Spearman correlation analysis between the 7 keystone taxa and the 21 functional biomarkers (Fig. 4H). A striking bipartite ecological network emerged. Pioneer taxa (e.g., *OTU_5* [*Staphylococcus*], *6* [*Bergeyella*], *10* [*unidentified_JGI_0000069-P22*], *12* [*Rothia*]) were strongly positively correlated with the declining aerobic pathways (e.g., *OTU_5* [*Staphylococcus*] vs. PWY-5896: *R* = 0.61, *p*_FDR_ < 0.001) but showed intense negative correlations with mature biofilm functions (Fig. 4H, Table S15). In stark contrast, the climax taxa (e.g., *OTU_17* [*Streptococcus*], *19* [*Staphylococcus*], *26* [*Kocuria*]) completely decoupled from early metabolic traits and exhibited robust positive correlations with late-stage pathways (Fig. 4H, Table S15). For instance, climax OTUs were highly correlated with peptidoglycan construction (*OTU_17* [*Streptococcus*] vs. PWY-6471: *R* = 0.75, *p*_FDR_ < 0.001), lactose degradation (*OTU_17* [*Streptococcus*] vs. LACTOSECAT-PWY: *R* = 0.72, *p*_FDR_ < 0.001), and sucrose degradation *(OTU_19* [*Staphylococcus*] vs. PWY-621: *R* = 0.33, *p*_FDR_ = 0.008, Fig. 4H, Table S15)). These strong correlative patterns suggest that the ecological handover from pioneer to climax species is closely linked to the metabolic specialization of the dental plaque.

### Ecological network topological evolution and keystone taxa trajectory during plaque biofilm maturation

**Fig. 5:**
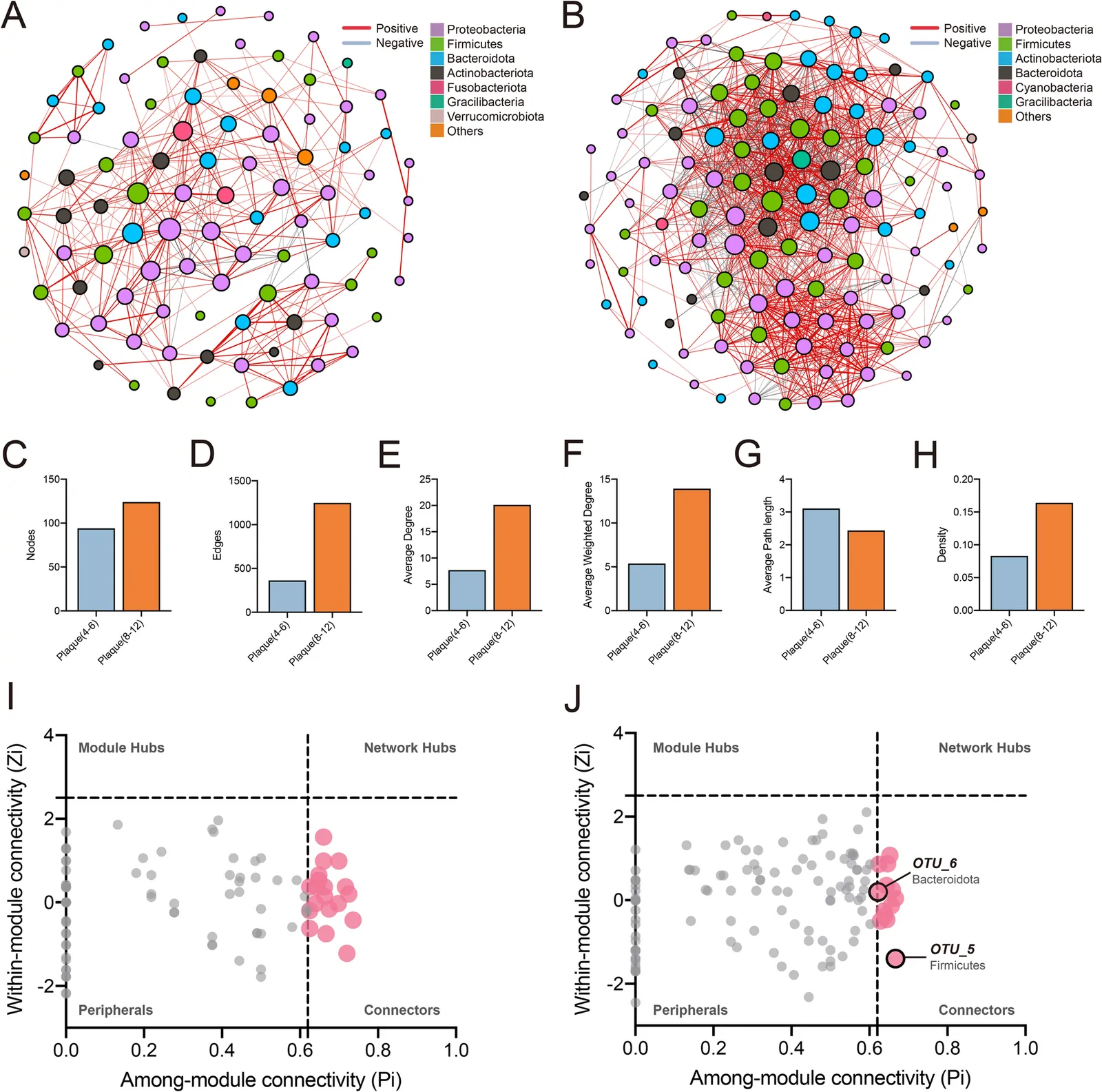
Ecological network topological evolution and keystone taxa analysis during early and mature stages of dental plaque biofilm. Microbial co-occurrence networks of dental plaque at (**A**) 4–6 months and (**B**) 8–12 months (|r| > 0.6, *p* < 0.01). Node color indicates Phylum-level taxonomy, and node size reflects degree weight. Red and blue edges represent positive and negative correlations, respectively. (**C**-**H**) Comparison of core topological parameters between the early (light blue) and mature (orange) networks. (**I**-**J**) Zi-Pi scatter plots showing the topological roles of nodes in the early and mature networks. Dashed lines indicate classical threshold boundaries, with nodes classified as inter-module Connectors highlighted in red

To evaluate the community interaction dynamics during dental plaque maturation, we constructed microbial co-occurrence networks for the early (4–6 months) and mature (8–12 months) developmental stages. Quantitative analysis of the network topological parameters confirmed a significant increase in community complexity and structural connectivity over time (Fig. 5A and B). Specifically, while the number of effective nodes exhibited a moderate increase from 94 in the early stage to 124 in the mature stage (Fig. 5C, Table S16), the number of significant interaction edges expanded explosively from 363 to 1,248 (Fig. 5D, Table S16). Consequently, the average degree and average weighted degree of the mature network reached 20.129 and 13.927, respectively, which were substantially higher than the 7.723 and 5.373 observed in the early network (Fig. 5E and F, Table S16). Furthermore, the average path length decreased from 3.107 to 2.437 (Fig. 5G, Table S16), whereas the overall network density doubled from 0.083 to 0.164 (Fig. 5H, Table S16). This combination of high density and shortened path length indicates that the mature dental plaque community developed a highly interconnected and structurally complex topology.

To further delineate the keystone taxa central to the network stability, we evaluated the within-module (Zi) and among-module (Pi) connectivity of all nodes in the early (Fig. 5I) and mature (Fig. 5J) networks. The Zi-Pi scatter plots revealed an absence of extreme network hubs or module hubs (Zi > 2.5) in both developmental stages, reflecting that the highly interconnected plaque biofilm does not rely on single dominant species, but rather on overall synergistic interactions. However, a critical set of inter-module connectors (Pi > 0.62, highlighted in red) emerged to maintain cross-module communication (Fig. 5I and J, Table S17, S18). Notably, in the mature network, *OTU_5* (*Staphylococcus*) and *OTU_6* (*Bergeyella*), which were previously identified as early pioneer species and whose absolute abundances had declined, strictly occupied the connector ecological niche (Fig. 5J, Table S17, S18). This quantitative topological shift indicates that early colonizers are not entirely outcompeted during biofilm maturation; instead, they transition into distinct connector niches, potentially mediating structural co-occurrences and supporting the integration of the climax community (e.g., Firmicutes) within the mature biofilm model.

## Discussion

The early assembly of the host-associated microbiome in highly specialized mammals represents a highly coordinated evolutionary process shaped by host ontogeny and environmental selection. By longitudinally tracking the spatial-temporal divergence of the oral microenvironment in giant panda cubs during their first year of life, this study demonstrates that the maturation of the dental plaque biofilm is not a stochastic accumulation of environmental microbes. Rather, it is a deterministic ecological succession driven by transient priority effects, autogenic niche reconstruction, and topological restructuring. These findings provide a theoretical framework for understanding how endangered herbivores establish resilient microecological barriers against extreme dietary transitions.

### Transient priority effects and autogenic niche reconstruction

Initial colonization within open microecosystems is frequently governed by priority effects, wherein early colonizers dictate subsequent successional trajectories through spatial preemption or resource depletion [40]. Our dynamic source-tracking model captured the transient nature of this phenomenon: at month 4, the contribution of historical saliva to dental plaque and the historical Mantel correlation simultaneously reached their peaks. This indicates that initial plaque colonization is strongly influenced by individual-specific historical imprints derived from early maternal contact and nursing, exhibiting a pronounced priority effect [41, 42]. Furthermore, although individual cubs inevitably received varying degrees of formula supplementation due to natural fluctuations in maternal milk production, our statistical framework (LMMs with individual random effects) robustly adjusted for these individualized dietary disparities. This suggests that the distinct ecological succession and niche differentiation observed herein are universally conserved developmental trajectories, rather than artifacts of specific formula interventions. Importantly, this robust statistical control also highlights a dual-layer assembly model. While the overarching structural restructuring and functional maturation of the plaque are deterministically driven by host ontogeny and tooth eruption, substantial individual-specific variation—a unique microbial fingerprint—persists at the baseline taxonomic level. As captured by our individual-constrained PERMANOVA and source-tracking models, this personalized variation is likely seeded by distinct maternal nursing behaviors and maintained by individual micro-environmental exposures, operating synchronously with the universal developmental pathways. However, this historical dominance rapidly attenuated by month 5, concomitant with a precipitous increase in the plaque-specific unknown fraction.

This rapid ecological turnover reflects profound physical and chemical differentiation within the oral microenvironment. Unlike shedding mucosal surfaces, the non-shedding hard surfaces of erupting teeth subject colonizing microbes to continuous hydrodynamic shear forces from salivary flow [17, 43]. Spatial preemption by planktonic salivary microbes is insufficient to resist this environmental filtering [44]. The compositional shift at month 5 marks a critical transition from reversible physical attachment to autogenic niche reconstruction [45, 46]. While a portion of the expanding “unknown fraction” may initially originate from transient environmental sources—introduced via the cubs’ frequent exploratory and wood-biting behaviors—their ability to persist and proliferate indicates successful adaptation. Consequently, this emergent fraction represents core benthic taxa capable of synthesizing extracellular polymeric substances (EPS) [47]. The progressive thickening of the biofilm matrix generates steep oxygen gradients, establishing a strictly anaerobic microenvironment that deterministically filters out early aerobic planktonic pioneers [48, 49]. Analogous paradigm shifts have been documented in human infant cohorts, where tooth eruption triggers the transition from a Streptococcus-dominated salivary community to a mature, Actinomyces- and Bacteroidetes-driven plaque biofilm [50]. This striking cross-species similarity highlights a fundamental ecological principle: while divergent diets and host genetics undoubtedly shape species-level specificities, the overarching successional trajectory is highly conserved. It suggests that the physical and anatomical changes driven by host ontogeny—specifically the emergence of non-shedding tooth surfaces and anaerobic crevices—act as primary, diet-independent deterministic filters. Consequently, these early colonizers perform conserved structural and ecological functions (e.g., biofilm scaffold construction and oxygen depletion) that lay a universal foundation for mature dental plaque, upon which diet subsequently acts as a secondary filter to fine-tune the community’s specialized metabolic repertoire.

### Host ontogeny is associated with microbial exchange and habitat mixing

Microbial succession is deeply coupled with host physiological developmental rhythms [51]. Concurrent Mantel tests revealed highly significant peaks in structural correlations between saliva and plaque at months 6 and 8. This transient cross-talk coincides with the concentrated eruption of deciduous teeth and the onset of exploratory mastication behaviors in giant panda cubs [52, 53]. From the perspective of saliva-plaque connectivity dynamics, high-frequency mechanical friction and masticatory stress act as intense ecological disturbances [54]. These physical shear forces continuously detach portions of the mature biofilm (the source), releasing them into the salivary flow (the sink), thereby causing a transient homogenization of the two habitats. Following this period of mechanical disruption, the communities diverged again between months 10 and 12. This re-differentiation indicates that the deeply stratified anaerobic biofilm had achieved structural maturation. The enhanced structural resilience of the climax community decouples it from the continuous fluctuations of the salivary environment, establishing a highly stable state [55, 56].

### Metabolic niche partitioning and microbiome-mediated evolutionary compensation

Taxonomic succession is inherently linked to metabolic niche partitioning [57]. Linear mixed-effects modeling demonstrated that mature plaque downregulates aerobic respiration pathways while specifically enriching for peptidoglycan biosynthesis and sucrose/lactose degradation. In the context of the giant panda’s evolutionary paradox, this functional transition extends beyond conventional biofilm maturation; it represents a critical microbiome-mediated evolutionary compensation [21, 58]. Because the host lacks the anatomical adaptations for prolonged retention and extensive hindgut fermentation of recalcitrant dietary fibers, the initial oral processing becomes the critical rate-limiting step for overall nutrient acquisition [8, 59]. Consequently, our observational findings suggest that the climax oral community may actively reinforce its physical barrier against intense masticatory shear forces while preemptively establishing a pre-adaptive metabolic reservoir for complex carbohydrate fermentation prior to the complete weaning transition [60]. Given the giant panda’s atypically short, carnivore-like gastrointestinal tract lacking extensive fermentation chambers, this initial oral processing is critical. We postulate that these polysaccharide-degrading oral taxa are continuously swallowed alongside the heavily masticated food bolus, acting as continuous microbial inoculants for the downstream digestive tract. By initiating the preliminary cleavage of complex carbohydrates, these oral-derived microbes likely synergize with the resident gut microbiota to facilitate the fermentation of dietary fibers into short-chain fatty acids (SCFAs, such as butyrate and acetate). This continuous microbial seeding from the oral cavity not only enhances overall digestive efficiency but also potentially contributes to the maintenance of intestinal epithelial health and immune homeostasis.

Within this competitive metabolic landscape, the developmental trajectory of early pioneer species (e.g., *OTU_5* [*Staphylococcus*] and *OTU_6* [*Bergeyella*]) elucidates both the underlying mechanisms of biofilm assembly and the topological resilience required for this evolutionary adaptation. According to the principle of competitive exclusion, pioneer taxa that are metabolically outcompeted during maturation should face local extinction [61]. However, topological network cartography revealed an alternative ecological strategy: despite a precipitous decline in absolute abundance, these pioneer species strictly occupied inter-module connector niches (Pi > 0.62) within the mature network. This finding fundamentally reframes the ecological role of pioneer taxa. Analogous to *Fusobacterium nucleatum* in mature human dental plaque—which functions as a structural scaffold mediating cross-taxon coaggregation between early Gram-positive and late Gram-negative colonizers, the pioneer species in the giant panda plaque likely undergo an ecological niche shift from metabolic drivers to structural bridging taxa [62]. By leveraging their broad coaggregation capabilities, they provide essential spatial scaffolds for the permanent attachment of late-arriving climax communities, particularly the Firmicutes [62]. This robust cross-taxon physical connectivity underpins the high metabolic synergy of the mature biofilm, ensuring that the oral microenvironment is structurally and functionally primed to endure the extreme biomechanical and metabolic stresses of the impending bamboo diet [58, 60].

### Implications, limitations, and future perspectives

Decoding the deterministic assembly rules of this highly specialized oral microenvironment not only advances our fundamental understanding of host-microbiome co-evolution and giant panda adaptive evolution, but also translates these theoretical insights into actionable support for preventive veterinary medicine. In captive environments, frequent exposure to dietary carbohydrates during the weaning transition often induces severe dental caries, which subsequently impairs adult bamboo foraging efficiency [9]. The climax taxa and network connectors identified in this study represent the foundational structural components of the native oral microbiome. These native keystone species offer highly precise biological targets for the development of host-specific oral probiotics, enabling targeted microecological interventions to maintain oral homeostasis during critical dietary shifts.

Methodological limitations of this study must be acknowledged. Amplicon sequencing of the 16 S rRNA gene provides taxonomic composition and predictive functional insights but lacks the resolution to identify specific virulence factors or coaggregation receptor expression profiles at the strain level [63]. Furthermore, inferences regarding metabolic reprogramming are based on genetic potential; integrating untargeted metabolomics is required to absolutely quantify cross-feeding networks of actual metabolites (e.g., short-chain fatty acids). Finally, this longitudinal cohort consisted exclusively of captive individuals. Given the distinct environmental microbial pools and maternal foraging behaviors experienced by wild populations, it is highly likely that wild panda cubs harbor a more diverse oral microbiota, seeded by constant exposure to heterogeneous forest environments. Therefore, while the fundamental ecological assembly rules (e.g., biofilm maturation driven by tooth eruption) are likely conserved, extrapolating these specific taxonomic diversity baselines to wild cubs requires ecological caution [45, 64, 65]. Future research should prioritize integrating multi-omics approaches and tracking individuals undergoing rewilding training to comprehensively decipher the microbiome-mediated adaptive evolution of the giant panda. The logical extension of this research is to longitudinally track the oral microbiome of juvenile giant pandas as they transition from weaning to active bamboo consumption. Investigating whether the climax communities and the “pre-adaptive metabolic reservoir” identified in this study are further recruited or functionally amplified upon the introduction of a bamboo-heavy diet will provide definitive insights into the microbiome-mediated adaptive evolution of the giant panda. Furthermore, future high-resolution longitudinal studies are warranted to quantify the microbial turnover rates and the stability of the mature climax community during the transition to adulthood.

## Conclusion

Our longitudinal investigation reframes the early-life assembly of the giant panda oral microbiome from a stochastic process to a deterministic mechanism of evolutionary compensation, mitigating the anatomical constraints of a carnivorous digestive tract facing an obligate herbivorous diet. Our results suggest that dental plaque maturation is characterized by a profound spatial-temporal divergence from saliva, wherein transient priority effects are rapidly superseded by autogenic niche reconstruction and pre-adaptive metabolic reprogramming for complex carbohydrate degradation. Concurrently, host ontogeny—notably deciduous tooth eruption—coincides with transient source-sink habitat homogenization, while topological network cartography reveals that early pioneer taxa evade competitive exclusion by structurally transitioning into inter-module connectors that robustly anchor late-arriving climax communities. Ultimately, deciphering these deterministic assembly rules and identifying native keystone scaffolds not only advances our fundamental understanding of host-microbiome co-evolution in specialized mammals, but also provides actionable, evolutionarily informed biological targets for preventive microecological interventions to safeguard vulnerable captive populations against diet-induced dental pathologies.

## Supplementary Information

Supplementary Material 1.

Supplementary Material 2.

## Data Availability

The raw amplicon sequencing data generated in this study have been deposited in the National Center for Biotechnology Information (NCBI) Sequence Read Archive (SRA) database. The 16 S rRNA gene sequences (V3–V4 regions) for the bacterial communities are publicly available under the BioProject accession number PRJNA1454587 (Saliva samples) and PRJNA1454200 (Plaque samples).
