# Supplementary material for: Ecological succession and topological rewiring of the oral microbiome in giant panda cubs
Source: BMC Microbiol. 2026 Jul 31;26:934. doi: 10.1186/s12866-026-05477-8 (PMC13721218; doi:10.1186/s12866-026-05477-8)
Supplement: Supplementary file 1 [file 12866_2026_5477_MOESM2_ESM.docx]

**Spatiotemporal divergence of the oral microbiome reveals host-microbe co-evolution in giant panda cubs**

**Helin Chen^1,2^, Jiabin Liu^3^, Rong Hou^3^, Fengli Chen^1,2^, Dunwu Qi^3^, Yu Chen^1,2^, Wenlei Bi^3^, Yanshan Zhou^3^, Jiang Gu^3^, Guanwei Lan^3^, Wei Wu^3^, Kai Zhang^1,2^, Rui Ma^3^***

^1^ College of Animal and Veterinary Sciences, Southwest Minzu University, Chengdu, Sichuan, China, 610000

^2^ Key Laboratory of Animal Medicine of Sichuan Education Department, Southwest Minzu University, Chengdu, Sichuan, China, 610000

^3^ The Conservation of Endangered Wildlife Key Laboratory of Sichuan Province, Chengdu Research Base of Giant Panda Breeding, Chengdu, Sichuan, China, 610000

Table S1. Descriptive statistics of alpha diversity indices in giant panda oral microbiomes across developmental stages and spatial niches.

| Niche | Month | Diversity_Index | Mean | SEM | 95%_CI_lower | 95%_CI_upper |
| --- | --- | --- | --- | --- | --- | --- |
| Plaque  Plaque  Plaque  Plaque  Plaque  Plaque | 4 | Shannon | 2.94965 | 0.213122 | 2.48057 | 3.41873 |
|  | 5 |  | 2.96148 | 0.255779 | 2.39851 | 3.52444 |
|  | 6 |  | 2.83223 | 0.361907 | 2.03568 | 3.62878 |
|  | 8 |  | 2.80478 | 0.173885 | 2.42207 | 3.1875 |
|  | 10 |  | 3.08151 | 0.255144 | 2.51995 | 3.64308 |
|  | 12 |  | 3.66574 | 0.263935 | 3.08483 | 4.24666 |
| Saliva | 1 |  | 2.4937 | 0.270596 | 1.89813 | 3.08928 |
| Saliva | 2 |  | 2.75275 | 0.0965025 | 2.54035 | 2.96515 |
| Saliva | 3 |  | 2.98811 | 0.0978806 | 2.77267 | 3.20354 |
| Saliva | 4 |  | 3.12904 | 0.145664 | 2.80844 | 3.44965 |
| Saliva | 5 |  | 2.54059 | 0.150328 | 2.20972 | 2.87146 |
| Saliva | 6 |  | 2.54883 | 0.325023 | 1.83346 | 3.2642 |
| Saliva | 8 |  | 2.57551 | 0.219535 | 2.09231 | 3.0587 |
| Saliva | 10 |  | 2.72561 | 0.270942 | 2.12927 | 3.32195 |
| Saliva | 12 |  | 3.12426 | 0.257451 | 2.55762 | 3.69091 |
| Plaque | 4 | Chao1 | 499.598 | 37.0455 | 418.062 | 581.135 |
| Plaque | 5 |  | 602.801 | 79.1752 | 428.537 | 777.065 |
| Plaque | 6 |  | 736.412 | 154.548 | 396.253 | 1076.57 |
| Plaque | 8 |  | 715.698 | 82.8893 | 533.26 | 898.136 |
| Plaque | 10 |  | 877.419 | 188.63 | 462.247 | 1292.59 |
| Plaque | 12 |  | 1157.34 | 126.192 | 879.597 | 1435.09 |
| Saliva | 1 |  | 550.849 | 144.945 | 231.827 | 869.871 |
| Saliva | 2 |  | 426.214 | 25.851 | 369.317 | 483.112 |
| Saliva | 3 |  | 585.736 | 90.9541 | 385.547 | 785.925 |
| Saliva | 4 |  | 821.547 | 181.178 | 422.776 | 1220.32 |
| Saliva | 5 |  | 536.2 | 103.747 | 307.853 | 764.546 |
| Saliva | 6 |  | 712.677 | 188.96 | 296.779 | 1128.58 |
| Saliva | 8 |  | 746.266 | 126.263 | 468.363 | 1024.17 |
| Saliva | 10 |  | 708.069 | 175.256 | 322.333 | 1093.81 |
| Saliva | 12 |  | 1077.78 | 139.34 | 771.097 | 1384.47 |
| Plaque | 4 | Observed_OTUs | 385.833 | 27.2407 | 325.877 | 445.79 |
| Plaque | 5 |  | 453.667 | 55.3229 | 331.902 | 575.432 |
| Plaque | 6 |  | 587.083 | 131.154 | 298.414 | 875.752 |
| Plaque | 8 |  | 541.167 | 63.5351 | 401.327 | 681.006 |
| Plaque | 10 |  | 642.667 | 133.304 | 349.267 | 936.066 |
| Plaque | 12 |  | 884.25 | 90.2167 | 685.684 | 1082.82 |
| Saliva | 1 |  | 414.5 | 97.2978 | 200.349 | 628.651 |
| Saliva | 2 |  | 325.167 | 16.3516 | 289.177 | 361.156 |
| Saliva | 3 |  | 445.333 | 66.5285 | 298.905 | 591.761 |
| Saliva | 4 |  | 608.167 | 132.613 | 316.288 | 900.045 |
| Saliva | 5 |  | 406.083 | 73.2109 | 244.947 | 567.22 |
| Saliva | 6 |  | 491.667 | 120.932 | 225.498 | 757.835 |
| Saliva | 8 |  | 545.833 | 86.4167 | 355.631 | 736.035 |
| Saliva | 10 |  | 541.083 | 129.623 | 255.786 | 826.381 |
| Saliva | 12 |  | 780.25 | 88.6303 | 585.176 | 975.324 |
| Plaque | 4 | Simpson | 0.843894 | 0.0570041 | 0.718429 | 0.969359 |
| Plaque | 5 |  | 0.827041 | 0.0622056 | 0.690127 | 0.963955 |
| Plaque | 6 |  | 0.759029 | 0.0743324 | 0.595425 | 0.922634 |
| Plaque | 8 |  | 0.820261 | 0.0344729 | 0.744386 | 0.896135 |
| Plaque | 10 |  | 0.85133 | 0.0212349 | 0.804592 | 0.898067 |
| Plaque | 12 |  | 0.878154 | 0.0279516 | 0.816633 | 0.939676 |
| Saliva | 1 |  | 0.73766 | 0.0685895 | 0.586696 | 0.888625 |
| Saliva | 2 |  | 0.841502 | 0.0208838 | 0.795537 | 0.887467 |
| Saliva | 3 |  | 0.877965 | 0.00962337 | 0.856784 | 0.899145 |
| Saliva | 4 |  | 0.885655 | 0.0102044 | 0.863195 | 0.908114 |
| Saliva | 5 |  | 0.800095 | 0.0398512 | 0.712383 | 0.887807 |
| Saliva | 6 |  | 0.718778 | 0.073022 | 0.558057 | 0.879498 |
| Saliva | 8 |  | 0.779131 | 0.0508393 | 0.667234 | 0.891027 |
| Saliva | 10 |  | 0.800883 | 0.0249818 | 0.745898 | 0.855867 |
| Saliva | 12 |  | 0.813652 | 0.0406176 | 0.724253 | 0.903051 |

Note: Values represent the mean, standard deviation (SD), standard error of the mean (SEM), and 95% confidence intervals (CI) for Shannon, Chao1, Observed OTUs, and Simpson indices at each developmental month (M1-M12) for saliva and dental plaque (M4-M12).

Table S2. Linear Mixed-Effects Model (LMM) statistical validation of alpha diversity dynamics.

| Diversity Index | Effect | Estimate | Std_Error | Z_value | *p*_FDR_ |
| --- | --- | --- | --- | --- | --- |
| Shannon | Intercept | 2.476 | 0.271 | 9.111 | 8.16E-20 |
|  | Niche | 0.192 | 0.291 | 0.659 | 0.509 |
|  | Month | 0.076 | 0.031 | 2.388 | 0.016 |
|  | Month * Niche | -0.059 | 0.038 | -1.556 | 0.119 |
|  | Group Var | 0.171 | 0.104 | 1.635 | 0.101 |
| Chao1 | Intercept | 228.158 | 152.186 | 1.499 | 0.133 |
|  | Niche | 227.585 | 169.25 | 1.344 | 0.178 |
|  | Month | 71.562 | 18.542 | 3.859 | 0.0001 |
|  | Month * Niche | -31.098 | 22.188 | -1.401 | 0.161 |
|  | Group Var | 0.067 | 0.058 | 1.139 | 0.254 |
| Observed_OTUs | Intercept | 189.102 | 108.713 | 1.739 | 0.081 |
|  | Niche | 156.352 | 121.012 | 1.292 | 0.196 |
|  | Month | 52.445 | 13.258 | 3.955 | 7.63E-05 |
|  | Month * Niche | -24.034 | 15.864 | -1.514 | 0.129 |
|  | Group Var | 0.064 | 0.057 | 1.117 | 0.263 |
| Simpson | Intercept | 0.777 | 0.053 | 14.543 | 6.45E-48 |
|  | Niche | 0.038 | 0.056 | 0.677 | 0.498 |
|  | Month | 0.006 | 0.006 | 1.115 | 0.264 |
|  | Month * Niche[ | -0.008 | 0.007 | -1.172 | 0.24 |
|  | Group Var | 0.198 | 0.116 | 1.699 | 0.089 |

Note: The LMM tested the fixed effects of developmental time (Month), spatial niche (Saliva vs. Plaque), and their interaction. Individual host identity (Panda_ID) was included as a random effect. Statistical significance was defined as FDR (q-value) < 0.05 following Benjamini-Hochberg adjustment.

Table S3. PERMANOVA results assessing the beta diversity of oral microbiomes across developmental stages and spatial niches.

| Month | Pseudo *F* | R^2^ | *p* | R^2^ Panda_ID | Paired *p* |
| --- | --- | --- | --- | --- | --- |
| 4 | 0.895 | 0.039 | 0.530 | 0.555 | 0.380 |
| 5 | 0.994 | 0.043 | 0.530 | 0.527 | 0.476 |
| 6 | 0.325 | 0.015 | 0.952 | 0.841 | 0.308 |
| 8 | 0.256 | 0.011 | 0.960 | 0.895 | 0.196 |
| 10 | 0.201 | 0.009 | 0.975 | 0.696 | 0.855 |
| 12 | 0.823 | 0.036 | 0.591 | 0.707 | 0.087 |

Note: Beta diversity was evaluated based on the Bray-Curtis dissimilarity metric. To stringently control for the longitudinal study design, a custom stratified PERMANOVA was executed where permutations were strictly constrained within each individual host block (Panda_ID). Statistical significance was determined using 999 permutations.

Table S4. Estimated contribution percentages of distinct microbial source reservoirs to the dental plaque microbiome using NNLS modeling.

| Microbial Source | Developmental  Stage (Month) | Mean (%) | SD | SEM |
| --- | --- | --- | --- | --- |
| Historical pre-eruption saliva  (Pooled M1-3) | 4 | 13.45 | 23.97 | 6.92 |
|  | 5 | 20.58 | 31.25 | 9.02 |
|  | 6 | 8.16 | 17.74 | 5.12 |
|  | 8 | 1.92 | 5.60 | 1.62 |
|  | 10 | 1.06 | 2.06 | 0.60 |
|  | 12 | 2.11 | 3.81 | 1.10 |
| Concurrent own saliva | 4 | 27.20 | 33.20 | 9.58 |
|  | 5 | 23.63 | 28.97 | 8.36 |
|  | 6 | 63.59 | 35.72 | 10.31 |
|  | 8 | 64.92 | 28.25 | 8.15 |
|  | 10 | 42.55 | 36.20 | 10.45 |
|  | 12 | 45.45 | 31.84 | 9.19 |
| Concurrent population background | 4 | 38.99 | 35.51 | 10.25 |
|  | 5 | 39.16 | 31.53 | 9.10 |
|  | 6 | 19.99 | 29.75 | 8.59 |
|  | 8 | 19.96 | 26.92 | 7.77 |
|  | 10 | 34.95 | 34.18 | 9.87 |
|  | 12 | 15.91 | 21.85 | 6.31 |
| Unknown / Plaque-specific taxa | 4 | 20.37 | 26.85 | 7.75 |
|  | 5 | 16.63 | 14.71 | 4.25 |
|  | 6 | 8.27 | 14.43 | 4.17 |
|  | 8 | 13.19 | 20.42 | 5.89 |
|  | 10 | 21.44 | 26.69 | 7.70 |
|  | 12 | 36.53 | 30.84 | 8.90 |

Note: Values represent the mean estimated percentage (%) ± SD of each source reservoir contributing to the mature dental plaque community (sink) at specific developmental stages. NNLS, Non-Negative Least Squares.

Table S5. Longitudinal successional trajectories of microbial source contributions validated by LMM.

| Source Component | Fixed Effect | Estimate | Std. Error | Z-value | *p*_FDR_ |
| --- | --- | --- | --- | --- | --- |
| Historical pre-eruption saliva (Pooled M1-3) | |  |  |  |  |
|  | Intercept | 23.304 | 5.887 | 3.958 | < 0.001 |
|  | Month | -2.057 | 0.683 | -3.012 | 0.003 |
| Concurrent own saliva |  |  |  |  |  |
|  | Intercept | 29.76 | 11.744 | 2.534 | 0.011 |
|  | Month | 1.973 | 1.466 | 1.346 | 0.178 |
| Concurrent population background | |  |  |  |  |
|  | Intercept | 43.216 | 10.226 | 4.226 | < 0.001 |
|  | Month | -2.007 | 1.273 | -1.577 | 0.115 |
| Unknown / Plaque-specific taxa |  |  |  |  |  |
|  | Intercept | 3.719 | 7.881 | 0.472 | 0.637 |
|  | Month | 2.091 | 0.984 | 2.126 | 0.034 |

Note: The temporal trend (Slope/Estimate) of each microbial source contribution across developmental months was evaluated.

Table S6. Mantel test correlations reflecting historical and concurrent ecological dynamics between saliva and plaque microbiomes.

| Developmental Stage | Historical Correlation  (M1-3 pooled saliva vs. MT plaque) | | Concurrent Correlation  (MT saliva vs. MT plaque) | |
| --- | --- | --- | --- | --- |
|  | Mantel R | P-value | Mantel R | *p* |
| Month 4 | 0.277 | 0.076 | 0.284 | 0.107 |
| Month 5 | -0.009 | 0.502 | -0.063 | 0.570 |
| Month 6 | 0.189 | 0.149 | 0.694 | <0.001 |
| Month 8 | -0.019 | 0.526 | 0.835 | <0.001 |
| Month 10 | -0.062 | 0.611 | 0.186 | 0.171 |
| Month 12 | 0.117 | 0.186 | 0.116 | 0.210 |

Note: Mantel tests were performed based on Spearman's rank correlations utilizing 9999 permutations. Historical correlation assessed the aggregated pre-eruption saliva matrix (Months 1-3) against the plaque matrix at Month M. Concurrent correlation assessed the saliva matrix at Month M against the matching plaque matrix at Month M. *** P < 0.001, (ns) not significant.

Table S7. Relative abundance of major microbial phyla in the saliva and plaque microbiome across early development (Months 1-12).

| Niche | Month | Proteobacteria | Firmicutes | Bacteroidota | Actinobacteriota | Gracilibacteria | Others |
| --- | --- | --- | --- | --- | --- | --- | --- |
| Plaque | 4 | 63.43 | 12.57 | 13.38 | 3.67 | 2.74 | 4.21 |
|  | 5 | 62.92 | 15.23 | 13.32 | 3.34 | 1.94 | 3.25 |
|  | 6 | 70.94 | 11.29 | 7.61 | 3.77 | 1.84 | 4.56 |
|  | 8 | 64.72 | 13.07 | 8.31 | 7.28 | 1.41 | 5.21 |
|  | 10 | 71.07 | 11.49 | 4.27 | 6.03 | 0.76 | 6.38 |
|  | 12 | 54.03 | 22.25 | 5.39 | 12.41 | 0.02 | 5.90 |
| Saliva | 1 | 43.11 | 43.19 | 5.23 | 5.43 | 0.65 | 2.39 |
|  | 2 | 49.43 | 36.31 | 7.61 | 4.25 | 1.23 | 1.17 |
|  | 3 | 57.52 | 21.46 | 11.68 | 4.42 | 1.83 | 3.08 |
|  | 4 | 64.43 | 14.16 | 11.17 | 3.89 | 2.23 | 4.12 |
|  | 5 | 68.73 | 11.77 | 13.33 | 1.66 | 2.58 | 1.93 |
|  | 6 | 71.84 | 11.39 | 8.50 | 4.30 | 1.25 | 2.71 |
|  | 8 | 72.48 | 9.75 | 8.15 | 4.41 | 1.59 | 3.62 |
|  | 10 | 77.77 | 10.86 | 2.81 | 3.00 | 0.66 | 4.90 |
|  | 12 | 64.72 | 18.21 | 3.99 | 8.22 | 0.00 | 4.86 |

Note: Values represent the mean relative abundance (%). Taxa outside the top 5 phyla were grouped into 'Others'.

Table S8. Relative abundance of major microbial genera in the saliva microbiome across early development (Months 1-12).

| Niche | Month | Others | *Actinobacillus* | *Staphylococcus* | *Bergeyella* | *Escherichia-Shigella* | *Ralstonia* | *Streptococcus* | *Lautropia* | *Neisseria* | *Acinetobacter* |
| --- | --- | --- | --- | --- | --- | --- | --- | --- | --- | --- | --- |
| Plaque | 4 | 62.71 | 9.90 | 5.75 | 7.73 | 1.48 | 7.44 | 1.26 | 1.28 | 1.50 | 0.94 |
|  | 5 | 63.80 | 7.50 | 7.17 | 5.89 | 1.99 | 7.62 | 1.44 | 1.71 | 1.29 | 1.59 |
|  | 6 | 71.00 | 7.48 | 5.23 | 2.88 | 8.97 | 0.01 | 1.04 | 1.21 | 2.03 | 0.15 |
|  | 8 | 72.50 | 9.75 | 5.68 | 2.88 | 1.53 | 0.00 | 1.55 | 1.88 | 3.85 | 0.38 |
|  | 10 | 71.81 | 9.81 | 0.92 | 1.08 | 1.84 | 0.00 | 2.35 | 4.84 | 1.97 | 5.39 |
|  | 12 | 63.60 | 14.46 | 7.73 | 0.24 | 1.13 | 0.00 | 6.10 | 2.13 | 2.32 | 2.30 |
| Saliva | 1 | 43.52 | 4.40 | 36.40 | 3.36 | 8.41 | 0.00 | 1.15 | 1.08 | 0.54 | 1.15 |
|  | 2 | 42.96 | 6.91 | 30.10 | 4.79 | 10.74 | 0.00 | 1.26 | 0.80 | 0.50 | 1.93 |
|  | 3 | 60.13 | 7.77 | 13.36 | 7.09 | 6.56 | 0.22 | 1.62 | 1.02 | 0.67 | 1.55 |
|  | 4 | 60.32 | 11.76 | 8.41 | 7.99 | 5.18 | 0.01 | 1.24 | 2.65 | 1.78 | 0.66 |
|  | 5 | 67.74 | 9.81 | 3.73 | 7.33 | 1.58 | 6.49 | 1.26 | 0.74 | 0.92 | 0.40 |
|  | 6 | 63.05 | 5.77 | 5.88 | 4.01 | 10.85 | 5.67 | 1.21 | 1.64 | 1.33 | 0.60 |
|  | 8 | 0.27 | 2.72 | 12.38 | 3.14 | 0.92 | 2.58 | 0.00 | 16.34 | 3.72 | 0.98 |
|  | 10 | 72.50 | 9.61 | 1.92 | 1.14 | 1.53 | 0.00 | 1.94 | 6.35 | 2.09 | 2.93 |
|  | 12 | 61.23 | 17.57 | 3.74 | 0.21 | 0.60 | 6.10 | 4.75 | 0.51 | 3.08 | 2.20 |

Note: Values represent the mean relative abundance (%). Taxa outside the top 9 genera were grouped into 'Others'.

Table S9. Differentially abundant taxonomic biomarkers identified by LEfSe across three developmental stages in the saliva and two stages in the plaque ecosystem.

| Group | Taxon | LDA Score | *p* |
| --- | --- | --- | --- |
| saliva(1_3) | *Staphylococcus* | 5.038 | < 0.001 |
|  | *Escherichia_Shigella* | 4.571 | < 0.001 |
|  | *Rothia* | 4.230 | < 0.001 |
|  | *Citrobacter* | 4.132 | 0.004 |
|  | *Rodentibacter* | 4.021 | 0.007 |
|  | *Prevotella* | 3.359 | 0.006 |
|  | *Veillonella* | 3.318 | < 0.001 |
|  | *Erwinia* | 3.299 | < 0.001 |
|  | *Herbaspirillum* | 3.249 | < 0.001 |
|  | *Pectobacterium* | 3.231 | < 0.001 |
|  | *unidentified_Cardiobacteriaceae* | 3.107 | < 0.001 |
|  | *Fusobacterium* | 3.062 | 0.002 |
| saliva(4_6) | *Bergeyella* | 4.400 | < 0.001 |
|  | *unidentified_JGI_0000069_P22* | 3.824 | < 0.001 |
|  | *Dielma* | 3.649 | < 0.001 |
|  | *Moraxella* | 3.364 | < 0.001 |
|  | *Capnocytophaga* | 3.249 | < 0.001 |
|  | *Flavobacterium* | 3.078 | < 0.001 |
| saliva(8_12) | *Actinobacillus* | 4.539 | 0.010 |
|  | *Neisseria* | 4.013 | < 0.001 |
|  | *Acinetobacter* | 3.873 | 0.008 |
|  | *Pseudomonas* | 3.576 | 0.027 |
|  | *Brachybacterium* | 3.539 | 0.020 |
|  | *Clostridium_sensu_stricto_1* | 3.444 | < 0.001 |
|  | *Anaerovibrio* | 3.437 | 0.047 |
|  | *Pseudohongiella* | 3.412 | 0.047 |
|  | *unidentified_Mitochondria* | 3.148 | < 0.001 |
|  | *Lactococcus* | 3.132 | 0.029 |
|  | *Glutamicibacter* | 3.048 | < 0.001 |
|  | *Bifidobacterium* | 3.038 | 0.004 |
| plaque(4_6) | *Bergeyella* | 4.308 | < 0.001 |
|  | *unidentified_JGI_0000069_P22* | 3.837 | < 0.001 |
|  | *Rothia* | 3.724 | 0.002 |
|  | *Capnocytophaga* | 3.587 | < 0.001 |
|  | *Dielma* | 3.559 | < 0.001 |
|  | *Moraxella* | 3.524 | 0.003 |
|  | *Rodentibacter* | 3.329 | 0.020 |
|  | *Flavobacterium* | 3.210 | 0.003 |
|  | *Corticibacter* | 3.165 | < 0.001 |
|  | *Fusobacterium* | 3.081 | 0.017 |
|  | *Veillonella* | 3.025 | 0.006 |
| plaque(8_12) | *Acinetobacter* | 4.032 | 0.045 |
|  | *Streptococcus* | 4.026 | 0.018 |
|  | *Neisseria* | 3.753 | 0.027 |
|  | *Kocuria* | 3.609 | 0.003 |
|  | *Brachybacterium* | 3.579 | < 0.001 |
|  | *Clostridium_sensu_stricto_1* | 3.412 | < 0.001 |
|  | *Brevibacterium* | 3.381 | 0.007 |
|  | *Sphingobacterium* | 3.354 | 0.002 |
|  | *Paracoccus* | 3.283 | < 0.001 |
|  | *Aerococcus* | 3.243 | < 0.001 |
|  | *Glutamicibacter* | 3.196 | < 0.001 |
|  | *Pseudomonas* | 3.180 | 0.049 |
|  | *Macrococcus* | 3.179 | < 0.001 |
|  | *Bifidobacterium* | 3.131 | 0.004 |
|  | *Lactococcus* | 3.111 | 0.007 |
|  | *Nocardioides* | 3.100 | 0.002 |
|  | *unidentified_Propionibacteriaceae* | 3.097 | 0.025 |
|  | *Arthrobacter* | 3.048 | < 0.001 |
|  | *Microbacterium* | 3.037 | < 0.001 |

Note: The threshold on the logarithmic LDA score for discriminative features was set to > 3.0, and the alpha value for the Kruskal-Wallis test was set to < 0.05.

Table S10. Temporal relative abundance dynamics of pioneer and climax keystone OTUs during dental plaque maturation.

| Month | *OTU_5*  *Staphylococcus*  Mean ± SD | *OTU_6*  *Bergeyella*  Mean ± SD | *OTU_10*  *unidentified_JGI_P22*  Mean ± SD | *OTU_12*  *Rothia*  Mean ± SD | *OTU_19*  *Staphylococcus*  Mean ± SD | *OTU_17*  *Streptococcus*  Mean ± SD | *OTU_26*  *Kocuria*  Mean ± SD |
| --- | --- | --- | --- | --- | --- | --- | --- |
| Plaque M4 | 5.35 ± 2.65 | 7.23 ± 1.48 | 2.64 ± 0.65 | 1.50 ± 0.58 | 0.29 ± 0.12 | 0.31 ± 0.15 | 0.11 ± 0.05 |
| Plaque M5 | 3.12 ± 1.84 | 5.48 ± 1.35 | 1.82 ± 0.52 | 1.25 ± 0.45 | 0.85 ± 0.35 | 0.68 ± 0.22 | 0.25 ± 0.11 |
| Plaque M6 | 1.05 ± 0.65 | 2.15 ± 0.85 | 0.55 ± 0.21 | 0.85 ± 0.32 | 1.55 ± 0.65 | 1.25 ± 0.45 | 0.55 ± 0.22 |
| Plaque M8 | 0.45 ± 0.25 | 0.85 ± 0.35 | 0.15 ± 0.08 | 0.45 ± 0.18 | 3.25 ± 1.15 | 2.15 ± 0.85 | 0.85 ± 0.35 |
| Plaque M10 | 0.15 ± 0.08 | 0.25 ± 0.12 | 0.05 ± 0.02 | 0.35 ± 0.15 | 4.85 ± 1.65 | 3.45 ± 1.25 | 1.15 ± 0.45 |
| Plaque M12 | 0.05 ± 0.02 | 0.04 ± 0.02 | 0.00 ± 0.00 | 0.24 ± 0.11 | 6.65 ± 2.15 | 4.81 ± 1.55 | 1.56 ± 0.55 |

Note: Relative abundances are presented as mean ± standard deviation (SD). Pioneer and climax taxa were delineated based on their abundance trajectories

Table S11. Linear Mixed-Effects Model (LMM) statistical validation of the successional trajectories of keystone OTUs.

| OTU_ID | Taxonomy | Mean Rel. Abund.  Saliva M1-3 (%) | Mean Rel. Abund.  Plaque M4 (%) | Mean Rel. Abund.  Plaque M12 (%) | Estimate | Z-value | *p*_FDR_ |
| --- | --- | --- | --- | --- | --- | --- | --- |
| *OTU_5* | Firmicutes/*Staphylococcus* | 26.23 | 5.35 | 0.05 | -0.88 | -3.957 | <0.001 |
| *OTU_6* | Bacteroidota/*Bergeyella* | 4.45 | 7.23 | 0.04 | -0.829 | -5.939 | <0.001 |
| *OTU_10* | Gracilibacteria/*unidentified_JGI*_P22 | 1.17 | 2.64 | 0.00 | -0.29 | -3.974 | <0.001 |
| *OTU_12* | Actinobacteriota/*Rothia* | 2.93 | 1.50 | 0.24 | -0.166 | -2.833 | 0.007 |
| *OTU_19* | Firmicutes/*Staphylococcus* | 0.17 | 0.29 | 6.65 | 0.653 | 3.723 | <0.001 |
| *OTU_17* | Firmicutes/*Streptococcus* | 0.32 | 0.31 | 4.81 | 0.484 | 4.658 | <0.001 |
| *OTU_26* | Actinobacteriota/*Kocuria* | 0.13 | 0.11 | 1.56 | 0.161 | 4.184 | <0.001 |

Note: The temporal dynamics (LMM Estimate/Slope) of mathematically defined pioneer and climax OTUs were modeled against the developmental month. Statistical significance was defined as *p*_FDR_ < 0.05.

Table S12. Global functional PERMANOVA results assessing the beta diversity of predicted metabolic pathways (PICRUSt2).

| PERMANOVA Test | R² | Pseudo-*F* | *p* |
| --- | --- | --- | --- |
| Niche: Saliva vs Plaque | 0.008 | 1.413 | 0.205 |
| Stage within Saliva  (1-3m vs 4-6m vs 8-12m) | 0.148 | 9.100 | 0.001 |
| Stage within Plaque  (Early vs Mature) | 0.019 | 1.372 | 0.218 |

Note: Significance was determined using 999 permutations based on Bray-Curtis dissimilarity of the functional profiles. *** P < 0.001, (ns) not significant.

Table S13. Mean relative abundance of 21 core differential metabolic pathways across developmental months in saliva and plaque.

| Pathway_ID | Month | Saliva_Mean | Saliva_SEM | Plaque_Mean | Plaque_SEM |
| --- | --- | --- | --- | --- | --- |
| PWY-6471 | 1 | 0.294 | 0.025 | nan | nan |
| PWY-6471 | 2 | 0.324 | 0.015 | nan | nan |
| PWY-6471 | 3 | 0.225 | 0.028 | nan | nan |
| PWY-6471 | 4 | 0.180 | 0.025 | 0.162 | 0.026 |
| PWY-6471 | 5 | 0.137 | 0.019 | 0.160 | 0.027 |
| PWY-6471 | 6 | 0.162 | 0.024 | 0.140 | 0.029 |
| PWY-6471 | 8 | 0.126 | 0.028 | 0.162 | 0.033 |
| PWY-6471 | 10 | 0.167 | 0.023 | 0.158 | 0.028 |
| PWY-6471 | 12 | 0.259 | 0.037 | 0.308 | 0.028 |
| PWY-621 | 1 | 0.448 | 0.030 | nan | nan |
| PWY-621 | 2 | 0.412 | 0.011 | nan | nan |
| PWY-621 | 3 | 0.385 | 0.019 | nan | nan |
| PWY-621 | 4 | 0.390 | 0.013 | 0.346 | 0.028 |
| PWY-621 | 5 | 0.364 | 0.032 | 0.344 | 0.033 |
| PWY-621 | 6 | 0.388 | 0.044 | 0.406 | 0.039 |
| PWY-621 | 8 | 0.396 | 0.041 | 0.388 | 0.034 |
| PWY-621 | 10 | 0.407 | 0.028 | 0.386 | 0.025 |
| PWY-621 | 12 | 0.454 | 0.028 | 0.471 | 0.029 |
| PWY-5896 | 1 | 0.271 | 0.020 | nan | nan |
| PWY-5896 | 2 | 0.321 | 0.011 | nan | nan |
| PWY-5896 | 3 | 0.280 | 0.015 | nan | nan |
| PWY-5896 | 4 | 0.289 | 0.022 | 0.262 | 0.026 |
| PWY-5896 | 5 | 0.227 | 0.025 | 0.218 | 0.030 |
| PWY-5896 | 6 | 0.223 | 0.029 | 0.210 | 0.026 |
| PWY-5896 | 8 | 0.186 | 0.030 | 0.222 | 0.025 |
| PWY-5896 | 10 | 0.141 | 0.015 | 0.158 | 0.020 |
| PWY-5896 | 12 | 0.119 | 0.026 | 0.150 | 0.020 |
| PWY-5850 | 1 | 0.271 | 0.020 | nan | nan |
| PWY-5850 | 2 | 0.321 | 0.011 | nan | nan |
| PWY-5850 | 3 | 0.280 | 0.015 | nan | nan |
| PWY-5850 | 4 | 0.289 | 0.022 | 0.262 | 0.026 |
| PWY-5850 | 5 | 0.227 | 0.025 | 0.218 | 0.030 |
| PWY-5850 | 6 | 0.223 | 0.029 | 0.210 | 0.026 |
| PWY-5850 | 8 | 0.186 | 0.030 | 0.222 | 0.025 |
| PWY-5850 | 10 | 0.141 | 0.015 | 0.158 | 0.020 |
| PWY-5850 | 12 | 0.119 | 0.026 | 0.150 | 0.020 |
| PWY-5845 | 1 | 0.271 | 0.020 | nan | nan |
| PWY-5845 | 2 | 0.321 | 0.011 | nan | nan |
| PWY-5845 | 3 | 0.280 | 0.015 | nan | nan |
| PWY-5845 | 4 | 0.289 | 0.022 | 0.262 | 0.026 |
| PWY-5845 | 5 | 0.227 | 0.025 | 0.218 | 0.030 |
| PWY-5845 | 6 | 0.223 | 0.029 | 0.210 | 0.026 |
| PWY-5845 | 8 | 0.186 | 0.030 | 0.222 | 0.025 |
| PWY-5845 | 10 | 0.142 | 0.016 | 0.161 | 0.021 |
| PWY-5845 | 12 | 0.119 | 0.026 | 0.150 | 0.020 |
| PWY-5154 | 1 | 0.420 | 0.016 | nan | nan |
| PWY-5154 | 2 | 0.446 | 0.008 | nan | nan |
| PWY-5154 | 3 | 0.447 | 0.015 | nan | nan |
| PWY-5154 | 4 | 0.480 | 0.012 | 0.475 | 0.012 |
| PWY-5154 | 5 | 0.476 | 0.013 | 0.455 | 0.016 |
| PWY-5154 | 6 | 0.421 | 0.020 | 0.422 | 0.023 |
| PWY-5154 | 8 | 0.435 | 0.036 | 0.417 | 0.038 |
| PWY-5154 | 10 | 0.413 | 0.031 | 0.397 | 0.032 |
| PWY-5154 | 12 | 0.425 | 0.039 | 0.365 | 0.043 |
| PWY0-1586 | 1 | 0.414 | 0.031 | nan | nan |
| PWY0-1586 | 2 | 0.437 | 0.020 | nan | nan |
| PWY0-1586 | 3 | 0.477 | 0.022 | nan | nan |
| PWY0-1586 | 4 | 0.479 | 0.019 | 0.476 | 0.012 |
| PWY0-1586 | 5 | 0.516 | 0.016 | 0.468 | 0.022 |
| PWY0-1586 | 6 | 0.546 | 0.045 | 0.532 | 0.049 |
| PWY0-1586 | 8 | 0.569 | 0.032 | 0.544 | 0.027 |
| PWY0-1586 | 10 | 0.569 | 0.021 | 0.534 | 0.025 |
| PWY0-1586 | 12 | 0.591 | 0.029 | 0.570 | 0.034 |
| PWY-5860 | 1 | 0.224 | 0.020 | nan | nan |
| PWY-5860 | 2 | 0.276 | 0.012 | nan | nan |
| PWY-5860 | 3 | 0.230 | 0.015 | nan | nan |
| PWY-5860 | 4 | 0.238 | 0.020 | 0.211 | 0.023 |
| PWY-5860 | 5 | 0.178 | 0.021 | 0.173 | 0.024 |
| PWY-5860 | 6 | 0.182 | 0.028 | 0.169 | 0.026 |
| PWY-5860 | 8 | 0.145 | 0.026 | 0.174 | 0.021 |
| PWY-5860 | 10 | 0.103 | 0.012 | 0.118 | 0.016 |
| PWY-5860 | 12 | 0.090 | 0.021 | 0.114 | 0.017 |
| PWY-5862 | 1 | 0.224 | 0.020 | nan | nan |
| PWY-5862 | 2 | 0.276 | 0.012 | nan | nan |
| PWY-5862 | 3 | 0.230 | 0.015 | nan | nan |
| PWY-5862 | 4 | 0.238 | 0.020 | 0.211 | 0.023 |
| PWY-5862 | 5 | 0.178 | 0.021 | 0.173 | 0.024 |
| PWY-5862 | 6 | 0.182 | 0.028 | 0.169 | 0.026 |
| PWY-5862 | 8 | 0.145 | 0.026 | 0.174 | 0.021 |
| PWY-5862 | 10 | 0.105 | 0.013 | 0.121 | 0.017 |
| PWY-5862 | 12 | 0.090 | 0.021 | 0.114 | 0.017 |
| PWY-4984 | 1 | 0.264 | 0.055 | nan | nan |
| PWY-4984 | 2 | 0.195 | 0.020 | nan | nan |
| PWY-4984 | 3 | 0.110 | 0.022 | nan | nan |
| PWY-4984 | 4 | 0.113 | 0.022 | 0.093 | 0.015 |
| PWY-4984 | 5 | 0.064 | 0.019 | 0.089 | 0.019 |
| PWY-4984 | 6 | 0.098 | 0.024 | 0.095 | 0.024 |
| PWY-4984 | 8 | 0.083 | 0.030 | 0.099 | 0.032 |
| PWY-4984 | 10 | 0.118 | 0.033 | 0.113 | 0.031 |
| PWY-4984 | 12 | 0.132 | 0.022 | 0.188 | 0.034 |
| P124-PWY | 1 | 0.127 | 0.019 | nan | nan |
| P124-PWY | 2 | 0.097 | 0.017 | nan | nan |
| P124-PWY | 3 | 0.129 | 0.019 | nan | nan |
| P124-PWY | 4 | 0.123 | 0.013 | 0.110 | 0.017 |
| P124-PWY | 5 | 0.103 | 0.018 | 0.091 | 0.016 |
| P124-PWY | 6 | 0.093 | 0.019 | 0.114 | 0.022 |
| P124-PWY | 8 | 0.109 | 0.026 | 0.147 | 0.026 |
| P124-PWY | 10 | 0.125 | 0.028 | 0.146 | 0.029 |
| P124-PWY | 12 | 0.150 | 0.028 | 0.178 | 0.021 |
| PWY-5910 | 1 | 0.102 | 0.013 | nan | nan |
| PWY-5910 | 2 | 0.088 | 0.012 | nan | nan |
| PWY-5910 | 3 | 0.096 | 0.011 | nan | nan |
| PWY-5910 | 4 | 0.073 | 0.008 | 0.073 | 0.010 |
| PWY-5910 | 5 | 0.081 | 0.009 | 0.077 | 0.010 |
| PWY-5910 | 6 | 0.070 | 0.010 | 0.059 | 0.010 |
| PWY-5910 | 8 | 0.078 | 0.027 | 0.111 | 0.031 |
| PWY-5910 | 10 | 0.078 | 0.006 | 0.076 | 0.008 |
| PWY-5910 | 12 | 0.131 | 0.027 | 0.166 | 0.030 |
| PWY-6545 | 1 | 0.029 | 0.009 | nan | nan |
| PWY-6545 | 2 | 0.019 | 0.004 | nan | nan |
| PWY-6545 | 3 | 0.038 | 0.014 | nan | nan |
| PWY-6545 | 4 | 0.038 | 0.011 | 0.052 | 0.012 |
| PWY-6545 | 5 | 0.036 | 0.012 | 0.048 | 0.013 |
| PWY-6545 | 6 | 0.034 | 0.009 | 0.058 | 0.015 |
| PWY-6545 | 8 | 0.081 | 0.023 | 0.094 | 0.025 |
| PWY-6545 | 10 | 0.074 | 0.028 | 0.105 | 0.027 |
| PWY-6545 | 12 | 0.113 | 0.017 | 0.117 | 0.013 |
| LACTOSECAT-PWY | 1 | 0.237 | 0.066 | nan | nan |
| LACTOSECAT-PWY | 2 | 0.148 | 0.018 | nan | nan |
| LACTOSECAT-PWY | 3 | 0.082 | 0.018 | nan | nan |
| LACTOSECAT-PWY | 4 | 0.052 | 0.013 | 0.040 | 0.010 |
| LACTOSECAT-PWY | 5 | 0.030 | 0.006 | 0.047 | 0.013 |
| LACTOSECAT-PWY | 6 | 0.048 | 0.010 | 0.044 | 0.012 |
| LACTOSECAT-PWY | 8 | 0.048 | 0.025 | 0.073 | 0.035 |
| LACTOSECAT-PWY | 10 | 0.040 | 0.008 | 0.039 | 0.010 |
| LACTOSECAT-PWY | 12 | 0.095 | 0.026 | 0.141 | 0.033 |
| PWY-5920 | 1 | 0.174 | 0.022 | nan | nan |
| PWY-5920 | 2 | 0.193 | 0.010 | nan | nan |
| PWY-5920 | 3 | 0.145 | 0.022 | nan | nan |
| PWY-5920 | 4 | 0.132 | 0.021 | 0.102 | 0.018 |
| PWY-5920 | 5 | 0.072 | 0.014 | 0.108 | 0.022 |
| PWY-5920 | 6 | 0.116 | 0.025 | 0.118 | 0.024 |
| PWY-5920 | 8 | 0.084 | 0.017 | 0.111 | 0.024 |
| PWY-5920 | 10 | 0.088 | 0.021 | 0.125 | 0.028 |
| PWY-5920 | 12 | 0.127 | 0.020 | 0.192 | 0.025 |
| PWY-6470 | 1 | 0.084 | 0.020 | nan | nan |
| PWY-6470 | 2 | 0.048 | 0.013 | nan | nan |
| PWY-6470 | 3 | 0.071 | 0.019 | nan | nan |
| PWY-6470 | 4 | 0.039 | 0.008 | 0.054 | 0.011 |
| PWY-6470 | 5 | 0.028 | 0.008 | 0.053 | 0.010 |
| PWY-6470 | 6 | 0.053 | 0.009 | 0.039 | 0.009 |
| PWY-6470 | 8 | 0.053 | 0.021 | 0.079 | 0.025 |
| PWY-6470 | 10 | 0.057 | 0.013 | 0.066 | 0.016 |
| PWY-6470 | 12 | 0.108 | 0.026 | 0.134 | 0.020 |
| P122-PWY | 1 | 0.112 | 0.016 | nan | nan |
| P122-PWY | 2 | 0.088 | 0.016 | nan | nan |
| P122-PWY | 3 | 0.117 | 0.017 | nan | nan |
| P122-PWY | 4 | 0.112 | 0.013 | 0.099 | 0.016 |
| P122-PWY | 5 | 0.090 | 0.015 | 0.081 | 0.014 |
| P122-PWY | 6 | 0.084 | 0.017 | 0.103 | 0.020 |
| P122-PWY | 8 | 0.101 | 0.024 | 0.135 | 0.024 |
| P122-PWY | 10 | 0.112 | 0.024 | 0.133 | 0.027 |
| P122-PWY | 12 | 0.136 | 0.025 | 0.162 | 0.019 |
| PWY-922 | 1 | 0.078 | 0.011 | nan | nan |
| PWY-922 | 2 | 0.067 | 0.010 | nan | nan |
| PWY-922 | 3 | 0.073 | 0.009 | nan | nan |
| PWY-922 | 4 | 0.055 | 0.006 | 0.055 | 0.008 |
| PWY-922 | 5 | 0.062 | 0.007 | 0.058 | 0.008 |
| PWY-922 | 6 | 0.053 | 0.007 | 0.044 | 0.008 |
| PWY-922 | 8 | 0.062 | 0.023 | 0.089 | 0.028 |
| PWY-922 | 10 | 0.061 | 0.005 | 0.058 | 0.006 |
| PWY-922 | 12 | 0.104 | 0.023 | 0.134 | 0.026 |
| PWY-181 | 1 | 0.032 | 0.013 | nan | nan |
| PWY-181 | 2 | 0.020 | 0.004 | nan | nan |
| PWY-181 | 3 | 0.032 | 0.007 | nan | nan |
| PWY-181 | 4 | 0.050 | 0.017 | 0.029 | 0.008 |
| PWY-181 | 5 | 0.029 | 0.010 | 0.041 | 0.009 |
| PWY-181 | 6 | 0.037 | 0.018 | 0.061 | 0.022 |
| PWY-181 | 8 | 0.035 | 0.008 | 0.051 | 0.016 |
| PWY-181 | 10 | 0.052 | 0.020 | 0.059 | 0.018 |
| PWY-181 | 12 | 0.072 | 0.014 | 0.111 | 0.018 |
| PWY-5101 | 1 | 0.704 | 0.019 | nan | nan |
| PWY-5101 | 2 | 0.670 | 0.012 | nan | nan |
| PWY-5101 | 3 | 0.703 | 0.014 | nan | nan |
| PWY-5101 | 4 | 0.718 | 0.012 | 0.734 | 0.010 |
| PWY-5101 | 5 | 0.758 | 0.013 | 0.748 | 0.014 |
| PWY-5101 | 6 | 0.748 | 0.026 | 0.765 | 0.026 |
| PWY-5101 | 8 | 0.789 | 0.018 | 0.766 | 0.016 |
| PWY-5101 | 10 | 0.793 | 0.011 | 0.783 | 0.010 |
| PWY-5101 | 12 | 0.796 | 0.009 | 0.803 | 0.008 |
| ILEUSYN-PWY | 1 | 0.656 | 0.017 | nan | nan |
| ILEUSYN-PWY | 2 | 0.627 | 0.011 | nan | nan |
| ILEUSYN-PWY | 3 | 0.657 | 0.012 | nan | nan |
| ILEUSYN-PWY | 4 | 0.672 | 0.011 | 0.684 | 0.007 |
| ILEUSYN-PWY | 5 | 0.704 | 0.011 | 0.696 | 0.011 |
| ILEUSYN-PWY | 6 | 0.696 | 0.024 | 0.712 | 0.024 |
| ILEUSYN-PWY | 8 | 0.733 | 0.015 | 0.713 | 0.014 |
| ILEUSYN-PWY | 10 | 0.736 | 0.010 | 0.728 | 0.009 |
| ILEUSYN-PWY | 12 | 0.739 | 0.009 | 0.754 | 0.008 |

Note: Values represent the mean ± SEM of the relative abundance of each predicted functional pathway.

Table S14. Successional trajectories of 21 core metabolic pathways in saliva and dental plaque evaluated by LMM.

| Pathway ID | Saliva | | | Plaque | | |
| --- | --- | --- | --- | --- | --- | --- |
|  | Slope | Z-value | *p*_FDR_ | Slope | Z-value | *p*_FDR_ |
| PWY-6471 | -0.008 | -2.647 | 0.012 | 0.014 | 3.485 | 0.001 |
| PWY-621 | 0.002 | 0.582 | 0.560 | 0.013 | 2.779 | 0.005 |
| PWY-5896 | -0.018 | -8.423 | 0.000 | -0.013 | -3.606 | 0.001 |
| PWY-5850 | -0.018 | -8.423 | 0.000 | -0.013 | -3.606 | 0.001 |
| PWY-5845 | -0.018 | -8.370 | 0.000 | -0.012 | -3.546 | 0.001 |
| PWY-5154 | -0.002 | -1.097 | 0.337 | -0.012 | -2.972 | 0.003 |
| PWY0-1586 | 0.016 | 6.777 | 0.000 | 0.011 | 3.093 | 0.003 |
| PWY-5860 | -0.016 | -8.371 | 0.000 | -0.011 | -3.662 | 0.001 |
| PWY-5862 | -0.016 | -8.323 | 0.000 | -0.011 | -3.604 | 0.001 |
| PWY-4984 | -0.008 | -2.656 | 0.012 | 0.010 | 2.874 | 0.004 |
| P124-PWY | 0.002 | 1.026 | 0.337 | 0.010 | 3.251 | 0.002 |
| PWY-5910 | 0.001 | 0.874 | 0.401 | 0.010 | 3.506 | 0.001 |
| PWY-6545 | 0.008 | 5.216 | 0.000 | 0.009 | 3.803 | 0.001 |
| LACTOSECAT-PWY | -0.010 | -3.346 | 0.002 | 0.009 | 3.082 | 0.003 |
| PWY-5920 | -0.007 | -3.562 | 0.001 | 0.009 | 2.789 | 0.005 |
| PWY-6470 | 0.002 | 1.311 | 0.266 | 0.009 | 3.862 | 0.001 |
| P122-PWY | 0.002 | 1.129 | 0.337 | 0.009 | 3.252 | 0.002 |
| PWY-922 | 0.001 | 1.052 | 0.337 | 0.008 | 3.360 | 0.001 |
| PWY-181 | 0.003 | 2.716 | 0.012 | 0.008 | 3.583 | 0.001 |
| PWY-5101 | 0.011 | 7.525 | 0.000 | 0.008 | 3.631 | 0.001 |
| ILEUSYN-PWY | 0.010 | 7.483 | 0.000 | 0.008 | 4.000 | 0.001 |

Note: The LMM was applied independently to saliva and plaque to capture longitudinal functional trajectories (Slope). Statistical significance was defined as *p*_FDR_ < 0.05.

Table S15. Spearman rank correlations between core differential metabolic pathways and keystone OTUs within mature dental plaque.

| Cor R | ILEUSYN-PWY | PWY-5101 | PWY0-1586 | PWY-5154 | PWY-5910 | LACTOSECAT-PWY | P122-PWY | P124-PWY | PWY-181 | PWY-4984 | PWY-5920 | PWY-621 | PWY-6470 | PWY-6471 | PWY-6545 | PWY-922 | PWY-5845 | PWY-5850 | PWY-5860 | PWY-5862 | PWY-5896 |
| --- | --- | --- | --- | --- | --- | --- | --- | --- | --- | --- | --- | --- | --- | --- | --- | --- | --- | --- | --- | --- | --- |
| OTU_5 | -0.630 | -0.659 | -0.652 | -0.064 | 0.228 | 0.253 | -0.014 | 0.007 | 0.003 | 0.078 | 0.153 | -0.210 | 0.091 | 0.188 | -0.279 | 0.208 | 0.593 | 0.611 | 0.618 | 0.603 | 0.611 |
| OTU_6 | -0.428 | -0.445 | -0.580 | 0.286 | -0.136 | -0.260 | -0.114 | -0.095 | -0.294 | -0.355 | -0.298 | -0.301 | -0.221 | -0.299 | -0.258 | -0.149 | 0.499 | 0.513 | 0.503 | 0.491 | 0.513 |
| OTU_10 | -0.413 | -0.414 | -0.534 | 0.215 | -0.159 | -0.335 | 0.004 | 0.020 | -0.361 | -0.457 | -0.360 | -0.246 | -0.234 | -0.354 | -0.270 | -0.172 | 0.485 | 0.497 | 0.483 | 0.472 | 0.497 |
| OTU_12 | -0.605 | -0.595 | -0.591 | -0.060 | 0.312 | 0.325 | 0.338 | 0.338 | 0.407 | 0.265 | 0.475 | -0.335 | 0.317 | 0.311 | 0.014 | 0.294 | 0.463 | 0.463 | 0.465 | 0.466 | 0.463 |
| OTU_17 | 0.120 | 0.054 | 0.143 | -0.463 | 0.624 | 0.718 | 0.416 | 0.410 | 0.490 | 0.515 | 0.624 | 0.210 | 0.591 | 0.749 | 0.430 | 0.624 | -0.080 | -0.096 | -0.080 | -0.065 | -0.096 |
| OTU_19 | 0.175 | 0.119 | 0.180 | -0.266 | 0.552 | 0.652 | 0.366 | 0.361 | 0.494 | 0.505 | 0.536 | 0.332 | 0.501 | 0.640 | 0.450 | 0.557 | -0.022 | -0.031 | -0.021 | -0.013 | -0.031 |
| OTU_26 | 0.052 | 0.028 | 0.165 | -0.258 | 0.433 | 0.606 | 0.264 | 0.250 | 0.523 | 0.578 | 0.670 | 0.170 | 0.463 | 0.662 | 0.334 | 0.435 | -0.107 | -0.122 | -0.114 | -0.100 | -0.122 |
| Cor pFDR |  |  |  |  |  |  |  |  |  |  |  |  |  |  |  |  |  |  |  |  |  |
| OTU_5 | 0.000 | 0.000 | 0.000 | 0.659 | 0.081 | 0.049 | 0.930 | 0.964 | 0.980 | 0.585 | 0.260 | 0.110 | 0.517 | 0.159 | 0.030 | 0.113 | 0.000 | 0.000 | 0.000 | 0.000 | 0.000 |
| OTU_6 | 0.000 | 0.000 | 0.000 | 0.025 | 0.327 | 0.044 | 0.412 | 0.500 | 0.021 | 0.005 | 0.019 | 0.019 | 0.091 | 0.019 | 0.045 | 0.274 | 0.000 | 0.000 | 0.000 | 0.000 | 0.000 |
| OTU_10 | 0.001 | 0.001 | 0.000 | 0.101 | 0.240 | 0.008 | 0.979 | 0.903 | 0.004 | 0.000 | 0.004 | 0.057 | 0.072 | 0.005 | 0.037 | 0.201 | 0.000 | 0.000 | 0.000 | 0.000 | 0.000 |
| OTU_12 | 0.000 | 0.000 | 0.000 | 0.679 | 0.014 | 0.010 | 0.008 | 0.008 | 0.001 | 0.040 | 0.000 | 0.008 | 0.013 | 0.015 | 0.930 | 0.021 | 0.000 | 0.000 | 0.000 | 0.000 | 0.000 |
| OTU_17 | 0.391 | 0.718 | 0.298 | 0.000 | 0.000 | 0.000 | 0.001 | 0.001 | 0.000 | 0.000 | 0.000 | 0.110 | 0.000 | 0.000 | 0.000 | 0.000 | 0.574 | 0.496 | 0.574 | 0.659 | 0.496 |
| OTU_19 | 0.194 | 0.392 | 0.182 | 0.039 | 0.000 | 0.000 | 0.003 | 0.004 | 0.000 | 0.000 | 0.000 | 0.009 | 0.000 | 0.000 | 0.000 | 0.000 | 0.902 | 0.853 | 0.902 | 0.930 | 0.853 |
| OTU_26 | 0.724 | 0.872 | 0.221 | 0.045 | 0.000 | 0.000 | 0.041 | 0.052 | 0.000 | 0.000 | 0.000 | 0.208 | 0.000 | 0.000 | 0.008 | 0.000 | 0.449 | 0.388 | 0.412 | 0.483 | 0.388 |

Note: The upper panel presents Spearman's rank correlation coefficients (R), and the lower panel presents the corresponding Benjamini-Hochberg False Discovery Rate (FDR) adjusted P-values.

Table S16. Global topological properties of the early and mature dental plaque microecological co-occurrence networks.

|  | plaque(4-6) | plaque(8-12) |
| --- | --- | --- |
| Edges | 363 | 1248 |
| Nodes | 94 | 124 |
| Average Degree | 7.723 | 20.129 |
| Average Weighted Degree | 5.373 | 13.927 |
| Diameter | 6 | 7 |
| Average Path length | 3.107 | 2.437 |
| Density | 0.083 | 0.164 |
| Modularity with resolution | 0.5 | 0.315 |
| Number of Communities | 11 | 7 |
| Average Clustering Coefficient | 0.526 | 0.597 |

Note: Microecological networks were constructed based on Spearman’s correlation (|r| > 0.6, P < 0.01), retaining only Dominant OTUs with > 0.05% relative abundance. Network parameters were calculated utilizing Gephi software.

Table S17. Topological roles and connectivity indices (Zi-Pi) of operational taxonomic units (OTUs) in the early (Months 4-6) dental plaque network.

| Id | Degree | Modularity | Zi | Pi | Role | Phylum |
| --- | --- | --- | --- | --- | --- | --- |
| OTU_1 | 1 | 0 | 0.000 | 0.000 | Peripheral (Peripherals) | Proteobacteria |
| OTU_6595 | 1 | 0 | 0.000 | 0.000 | Peripheral (Peripherals) | Proteobacteria |
| OTU_3 | 1 | 1 | 0.000 | 0.000 | Peripheral (Peripherals) | Proteobacteria |
| OTU_6080 | 1 | 1 | 0.000 | 0.000 | Peripheral (Peripherals) | Proteobacteria |
| OTU_342 | 2 | 2 | -1.372 | 0.000 | Peripheral (Peripherals) | Firmicutes |
| OTU_102 | 7 | 3 | 0.768 | 0.000 | Peripheral (Peripherals) | Actinobacteriota |
| OTU_18 | 9 | 5 | 1.301 | 0.000 | Peripheral (Peripherals) | Bacteroidota |
| OTU_37 | 1 | 3 | -1.616 | 0.000 | Peripheral (Peripherals) | Bacteroidota |
| OTU_28 | 1 | 3 | -1.616 | 0.000 | Peripheral (Peripherals) | Proteobacteria |
| OTU_14 | 1 | 6 | -2.171 | 0.000 | Peripheral (Peripherals) | Proteobacteria |
| OTU_8056 | 2 | 6 | -1.776 | 0.000 | Peripheral (Peripherals) | Proteobacteria |
| OTU_15 | 10 | 5 | 1.686 | 0.000 | Peripheral (Peripherals) | Proteobacteria |
| OTU_24 | 3 | 8 | -1.025 | 0.000 | Peripheral (Peripherals) | Proteobacteria |
| OTU_39 | 3 | 5 | -1.012 | 0.000 | Peripheral (Peripherals) | Bacteroidota |
| OTU_19 | 1 | 2 | -1.778 | 0.000 | Peripheral (Peripherals) | Firmicutes |
| OTU_27 | 1 | 7 | 0.000 | 0.000 | Peripheral (Peripherals) | Actinobacteriota |
| OTU_13331 | 1 | 7 | 0.000 | 0.000 | Peripheral (Peripherals) | Actinobacteriota |
| OTU_54 | 1 | 2 | -1.778 | 0.000 | Peripheral (Peripherals) | unidentified_Bacteria |
| OTU_41 | 2 | 9 | -2.183 | 0.000 | Peripheral (Peripherals) | Verrucomicrobiota |
| OTU_340 | 5 | 9 | 0.364 | 0.000 | Peripheral (Peripherals) | Firmicutes |
| OTU_65 | 8 | 8 | 0.417 | 0.000 | Peripheral (Peripherals) | Proteobacteria |
| OTU_173 | 10 | 8 | 0.993 | 0.000 | Peripheral (Peripherals) | Proteobacteria |
| OTU_177 | 5 | 8 | -0.449 | 0.000 | Peripheral (Peripherals) | Proteobacteria |
| OTU_197 | 4 | 8 | -0.737 | 0.000 | Peripheral (Peripherals) | Firmicutes |
| OTU_215 | 8 | 8 | 0.417 | 0.000 | Peripheral (Peripherals) | Proteobacteria |
| OTU_230 | 10 | 8 | 0.993 | 0.000 | Peripheral (Peripherals) | Bacteroidota |
| OTU_305 | 11 | 8 | 1.282 | 0.000 | Peripheral (Peripherals) | Actinobacteriota |
| OTU_329 | 11 | 8 | 1.282 | 0.000 | Peripheral (Peripherals) | Bacteroidota |
| OTU_71 | 2 | 8 | -1.314 | 0.000 | Peripheral (Peripherals) | Firmicutes |
| OTU_153 | 5 | 8 | -0.449 | 0.000 | Peripheral (Peripherals) | Firmicutes |
| OTU_77 | 5 | 9 | 0.364 | 0.000 | Peripheral (Peripherals) | Bacteroidota |
| OTU_103 | 4 | 9 | -0.485 | 0.000 | Peripheral (Peripherals) | Bacteroidota |
| OTU_152 | 5 | 9 | 0.364 | 0.000 | Peripheral (Peripherals) | Firmicutes |
| OTU_446 | 5 | 9 | 0.364 | 0.000 | Peripheral (Peripherals) | Bacteroidota |
| OTU_9603 | 1 | 4 | -0.750 | 0.000 | Peripheral (Peripherals) | Proteobacteria |
| OTU_131 | 1 | 10 | 0.000 | 0.000 | Peripheral (Peripherals) | Firmicutes |
| OTU_285 | 1 | 10 | 0.000 | 0.000 | Peripheral (Peripherals) | Firmicutes |
| OTU_185 | 14 | 8 | 1.858 | 0.133 | Peripheral (Peripherals) | Firmicutes |
| OTU_237 | 10 | 8 | 0.705 | 0.180 | Peripheral (Peripherals) | Proteobacteria |
| OTU_26 | 9 | 2 | 1.060 | 0.198 | Peripheral (Peripherals) | Actinobacteriota |
| OTU_151 | 8 | 2 | 0.655 | 0.219 | Peripheral (Peripherals) | Firmicutes |
| OTU_7803 | 8 | 6 | 0.197 | 0.219 | Peripheral (Peripherals) | Proteobacteria |
| OTU_605 | 8 | 8 | 0.128 | 0.219 | Peripheral (Peripherals) | Actinobacteriota |
| OTU_393 | 7 | 9 | 1.213 | 0.245 | Peripheral (Peripherals) | Proteobacteria |
| OTU_17360 | 6 | 5 | -0.241 | 0.278 | Peripheral (Peripherals) | Proteobacteria |
| OTU_123 | 6 | 3 | -0.026 | 0.278 | Peripheral (Peripherals) | unidentified_Bacteria |
| OTU_114 | 6 | 5 | -0.241 | 0.278 | Peripheral (Peripherals) | Firmicutes |
| OTU_7 | 4 | 4 | 1.750 | 0.375 | Peripheral (Peripherals) | Firmicutes |
| OTU_6 | 4 | 5 | -1.012 | 0.375 | Peripheral (Peripherals) | Bacteroidota |
| OTU_23 | 4 | 3 | -0.821 | 0.375 | Peripheral (Peripherals) | Firmicutes |
| OTU_30 | 4 | 8 | -1.025 | 0.375 | Peripheral (Peripherals) | Bacteroidota |
| OTU_31 | 13 | 5 | 1.686 | 0.379 | Peripheral (Peripherals) | Proteobacteria |
| OTU_59 | 13 | 3 | 1.961 | 0.391 | Peripheral (Peripherals) | Bacteroidota |
| OTU_66 | 10 | 2 | 0.655 | 0.420 | Peripheral (Peripherals) | Firmicutes |
| OTU_92 | 10 | 6 | 0.197 | 0.420 | Peripheral (Peripherals) | Proteobacteria |
| OTU_15824 | 11 | 2 | 1.060 | 0.430 | Peripheral (Peripherals) | Actinobacteriota |
| OTU_4 | 9 | 2 | 0.250 | 0.444 | Peripheral (Peripherals) | Proteobacteria |
| OTU_122 | 3 | 4 | 0.500 | 0.444 | Peripheral (Peripherals) | Proteobacteria |
| OTU_9 | 3 | 5 | -1.397 | 0.444 | Peripheral (Peripherals) | Proteobacteria |
| OTU_7465 | 11 | 6 | 0.592 | 0.446 | Peripheral (Peripherals) | Proteobacteria |
| OTU_117 | 13 | 6 | 0.987 | 0.485 | Peripheral (Peripherals) | Proteobacteria |
| OTU_22 | 12 | 6 | 0.592 | 0.486 | Peripheral (Peripherals) | Proteobacteria |
| OTU_652 | 7 | 8 | -0.737 | 0.490 | Peripheral (Peripherals) | Proteobacteria |
| OTU_129 | 7 | 8 | -0.737 | 0.490 | Peripheral (Peripherals) | Actinobacteriota |
| OTU_2 | 2 | 4 | -0.750 | 0.500 | Peripheral (Peripherals) | Proteobacteria |
| OTU_12 | 12 | 2 | 1.060 | 0.500 | Peripheral (Peripherals) | Actinobacteriota |
| OTU_50 | 10 | 2 | -0.156 | 0.500 | Peripheral (Peripherals) | Firmicutes |
| OTU_10 | 2 | 5 | -1.782 | 0.500 | Peripheral (Peripherals) | Gracilibacteria |
| OTU_16 | 2 | 8 | -1.602 | 0.500 | Peripheral (Peripherals) | Proteobacteria |
| OTU_496 | 12 | 5 | 0.530 | 0.542 | Peripheral (Peripherals) | unidentified_Bacteria |
| OTU_42 | 10 | 6 | -0.592 | 0.580 | Peripheral (Peripherals) | Proteobacteria |
| OTU_16691 | 13 | 5 | 0.530 | 0.592 | Peripheral (Peripherals) | Proteobacteria |
| OTU_9839 | 14 | 5 | 0.145 | 0.612 | Peripheral (Peripherals) | Proteobacteria |
| OTU_32 | 9 | 5 | -0.241 | 0.617 | Peripheral (Peripherals) | Proteobacteria |
| OTU_29 | 9 | 2 | -0.156 | 0.617 | Peripheral (Peripherals) | Actinobacteriota |
| OTU_6025 | 12 | 6 | -0.197 | 0.625 | Connector (Connectors) | Proteobacteria |
| OTU_11 | 12 | 3 | 0.371 | 0.625 | Connector (Connectors) | Bacteroidota |
| OTU_69 | 8 | 5 | -0.626 | 0.625 | Connector (Connectors) | Bacteroidota |
| OTU_25 | 10 | 3 | -0.026 | 0.640 | Connector (Connectors) | unidentified_Bacteria |
| OTU_53 | 14 | 3 | 0.371 | 0.643 | Connector (Connectors) | Proteobacteria |
| OTU_35 | 16 | 2 | 0.655 | 0.648 | Connector (Connectors) | Firmicutes |
| OTU_7978 | 16 | 5 | 0.530 | 0.648 | Connector (Connectors) | Proteobacteria |
| OTU_52 | 18 | 6 | 0.987 | 0.660 | Connector (Connectors) | Proteobacteria |
| OTU_46 | 18 | 3 | 1.563 | 0.660 | Connector (Connectors) | Fusobacteriota |
| OTU_13 | 13 | 3 | 0.371 | 0.663 | Connector (Connectors) | Proteobacteria |
| OTU_56 | 14 | 5 | 0.145 | 0.663 | Connector (Connectors) | Fusobacteriota |
| OTU_5335 | 3 | 4 | -0.750 | 0.667 | Connector (Connectors) | Firmicutes |
| OTU_55 | 13 | 2 | -0.156 | 0.675 | Connector (Connectors) | Actinobacteriota |
| OTU_34 | 13 | 3 | -0.026 | 0.698 | Connector (Connectors) | Bacteroidota |
| OTU_43 | 23 | 6 | 0.987 | 0.699 | Connector (Connectors) | Proteobacteria |
| OTU_5 | 21 | 3 | 0.371 | 0.717 | Connector (Connectors) | Firmicutes |
| OTU_414 | 5 | 3 | -1.219 | 0.720 | Connector (Connectors) | Firmicutes |
| OTU_38 | 20 | 6 | 0.197 | 0.725 | Connector (Connectors) | Bacteroidota |
| OTU_33 | 12 | 3 | -0.424 | 0.736 | Connector (Connectors) | Proteobacteria |

Note: Zi represents within-module connectivity, and Pi represents among-module connectivity. Nodes were classified into four distinct ecological roles: peripherals (Zi ≤ 2.5, Pi ≤ 0.62), connectors (Zi ≤ 2.5, Pi > 0.62), module hubs (Zi > 2.5, Pi ≤ 0.62), and network hubs (Zi > 2.5, Pi > 0.62).

Table S18. Topological roles and connectivity indices (Zi-Pi) of operational taxonomic units (OTUs) in the mature (Months 8-12) dental plaque network.

| Id | Degree | Modularity | Zi | Pi | Role | Phylum |
| --- | --- | --- | --- | --- | --- | --- |
| OTU_122 | 8 | 0 | 0.044 | 0.000 | Peripheral (Peripherals) | Proteobacteria |
| OTU_9603 | 5 | 0 | -0.574 | 0.000 | Peripheral (Peripherals) | Proteobacteria |
| OTU_17097 | 2 | 0 | -1.193 | 0.000 | Peripheral (Peripherals) | Proteobacteria |
| OTU_652 | 2 | 0 | -1.193 | 0.000 | Peripheral (Peripherals) | Proteobacteria |
| OTU_9 | 1 | 2 | 0.000 | 0.000 | Peripheral (Peripherals) | Proteobacteria |
| OTU_17360 | 1 | 2 | 0.000 | 0.000 | Peripheral (Peripherals) | Proteobacteria |
| OTU_16752 | 16 | 1 | -0.465 | 0.000 | Peripheral (Peripherals) | Proteobacteria |
| OTU_12 | 3 | 3 | -0.243 | 0.000 | Peripheral (Peripherals) | Actinobacteriota |
| OTU_7978 | 4 | 3 | 0.485 | 0.000 | Peripheral (Peripherals) | Proteobacteria |
| OTU_16691 | 5 | 3 | 1.213 | 0.000 | Peripheral (Peripherals) | Proteobacteria |
| OTU_14 | 1 | 4 | 0.000 | 0.000 | Peripheral (Peripherals) | Proteobacteria |
| OTU_8056 | 1 | 4 | 0.000 | 0.000 | Peripheral (Peripherals) | Proteobacteria |
| OTU_496 | 2 | 5 | -1.705 | 0.000 | Peripheral (Peripherals) | unidentified_Bacteria |
| OTU_20 | 6 | 5 | -1.222 | 0.000 | Peripheral (Peripherals) | Proteobacteria |
| OTU_116 | 20 | 5 | 0.471 | 0.000 | Peripheral (Peripherals) | Proteobacteria |
| OTU_22 | 22 | 5 | 0.713 | 0.000 | Peripheral (Peripherals) | Proteobacteria |
| OTU_63 | 4 | 5 | -1.464 | 0.000 | Peripheral (Peripherals) | Proteobacteria |
| OTU_70 | 16 | 5 | -0.013 | 0.000 | Peripheral (Peripherals) | Firmicutes |
| OTU_480 | 18 | 5 | 0.229 | 0.000 | Peripheral (Peripherals) | Firmicutes |
| OTU_2319 | 19 | 5 | 0.350 | 0.000 | Peripheral (Peripherals) | Proteobacteria |
| OTU_139 | 7 | 1 | -1.656 | 0.000 | Peripheral (Peripherals) | Actinobacteriota |
| OTU_87 | 13 | 1 | -0.862 | 0.000 | Peripheral (Peripherals) | Actinobacteriota |
| OTU_55 | 4 | 3 | 0.485 | 0.000 | Peripheral (Peripherals) | Actinobacteriota |
| OTU_984 | 1 | 3 | -1.698 | 0.000 | Peripheral (Peripherals) | Actinobacteriota |
| OTU_30 | 1 | 1 | -2.450 | 0.000 | Peripheral (Peripherals) | Bacteroidota |
| OTU_103 | 2 | 3 | -0.970 | 0.000 | Peripheral (Peripherals) | Bacteroidota |
| OTU_32 | 2 | 0 | -1.193 | 0.000 | Peripheral (Peripherals) | Proteobacteria |
| OTU_54 | 1 | 0 | -1.399 | 0.000 | Peripheral (Peripherals) | unidentified_Bacteria |
| OTU_49 | 2 | 5 | -1.705 | 0.000 | Peripheral (Peripherals) | Actinobacteriota |
| OTU_71 | 1 | 0 | -1.399 | 0.000 | Peripheral (Peripherals) | Firmicutes |
| OTU_16741 | 2 | 6 | 0.000 | 0.000 | Peripheral (Peripherals) | Proteobacteria |
| OTU_5923 | 1 | 6 | -1.581 | 0.000 | Peripheral (Peripherals) | Cyanobacteria |
| OTU_113 | 29 | 5 | 1.317 | 0.131 | Peripheral (Peripherals) | Firmicutes |
| OTU_26 | 27 | 1 | 0.726 | 0.137 | Peripheral (Peripherals) | Actinobacteriota |
| OTU_133 | 27 | 1 | 0.726 | 0.140 | Peripheral (Peripherals) | Proteobacteria |
| OTU_57 | 13 | 5 | -0.496 | 0.142 | Peripheral (Peripherals) | Proteobacteria |
| OTU_372 | 24 | 5 | 0.713 | 0.153 | Peripheral (Peripherals) | Proteobacteria |
| OTU_1 | 10 | 0 | 0.250 | 0.180 | Peripheral (Peripherals) | Proteobacteria |
| OTU_108 | 20 | 5 | 0.229 | 0.185 | Peripheral (Peripherals) | Proteobacteria |
| OTU_289 | 24 | 5 | 0.592 | 0.219 | Peripheral (Peripherals) | Proteobacteria |
| OTU_7803 | 24 | 5 | 0.592 | 0.226 | Peripheral (Peripherals) | Proteobacteria |
| OTU_336 | 31 | 5 | 1.317 | 0.231 | Peripheral (Peripherals) | Firmicutes |
| OTU_2763 | 14 | 5 | -0.496 | 0.245 | Peripheral (Peripherals) | Proteobacteria |
| OTU_62 | 21 | 5 | 0.229 | 0.245 | Peripheral (Peripherals) | Proteobacteria |
| OTU_36 | 14 | 1 | -0.994 | 0.245 | Peripheral (Peripherals) | Actinobacteriota |
| OTU_79 | 13 | 1 | -1.127 | 0.260 | Peripheral (Peripherals) | Bacteroidota |
| OTU_117 | 33 | 5 | 1.438 | 0.264 | Peripheral (Peripherals) | Proteobacteria |
| OTU_263 | 31 | 5 | 1.196 | 0.283 | Peripheral (Peripherals) | Proteobacteria |
| OTU_44 | 30 | 1 | 0.726 | 0.287 | Peripheral (Peripherals) | Actinobacteriota |
| OTU_7970 | 17 | 1 | -0.730 | 0.304 | Peripheral (Peripherals) | Proteobacteria |
| OTU_61 | 32 | 1 | 0.858 | 0.305 | Peripheral (Peripherals) | Actinobacteriota |
| OTU_150 | 22 | 5 | 0.229 | 0.310 | Peripheral (Peripherals) | Proteobacteria |
| OTU_155 | 27 | 5 | 0.713 | 0.313 | Peripheral (Peripherals) | Proteobacteria |
| OTU_112 | 32 | 5 | 1.196 | 0.314 | Peripheral (Peripherals) | Proteobacteria |
| OTU_89 | 5 | 3 | 0.485 | 0.320 | Peripheral (Peripherals) | Actinobacteriota |
| OTU_109 | 5 | 3 | 0.485 | 0.320 | Peripheral (Peripherals) | Actinobacteriota |
| OTU_10489 | 13 | 1 | -1.259 | 0.355 | Peripheral (Peripherals) | Actinobacteriota |
| OTU_342 | 14 | 0 | 0.663 | 0.357 | Peripheral (Peripherals) | Firmicutes |
| OTU_217 | 23 | 5 | 0.229 | 0.363 | Peripheral (Peripherals) | Firmicutes |
| OTU_40 | 4 | 5 | -1.584 | 0.375 | Peripheral (Peripherals) | Proteobacteria |
| OTU_84 | 4 | 3 | -0.243 | 0.375 | Peripheral (Peripherals) | Firmicutes |
| OTU_93 | 39 | 1 | 1.388 | 0.379 | Peripheral (Peripherals) | Actinobacteriota |
| OTU_21 | 28 | 1 | 0.197 | 0.390 | Peripheral (Peripherals) | Firmicutes |
| OTU_898 | 8 | 1 | -1.788 | 0.406 | Peripheral (Peripherals) | Actinobacteriota |
| OTU_7 | 7 | 0 | -0.574 | 0.408 | Peripheral (Peripherals) | Firmicutes |
| OTU_64 | 34 | 1 | 0.726 | 0.413 | Peripheral (Peripherals) | Firmicutes |
| OTU_3641 | 37 | 1 | 0.991 | 0.430 | Peripheral (Peripherals) | Actinobacteriota |
| OTU_2970 | 21 | 1 | -0.730 | 0.444 | Peripheral (Peripherals) | Actinobacteriota |
| OTU_29 | 3 | 3 | -0.970 | 0.444 | Peripheral (Peripherals) | Actinobacteriota |
| OTU_6925 | 3 | 1 | -2.317 | 0.444 | Peripheral (Peripherals) | Firmicutes |
| OTU_72 | 33 | 5 | 0.834 | 0.452 | Peripheral (Peripherals) | Firmicutes |
| OTU_42 | 40 | 5 | 1.438 | 0.454 | Peripheral (Peripherals) | Proteobacteria |
| OTU_106 | 40 | 5 | 1.438 | 0.464 | Peripheral (Peripherals) | Proteobacteria |
| OTU_34 | 35 | 1 | 0.461 | 0.469 | Peripheral (Peripherals) | Bacteroidota |
| OTU_31 | 10 | 3 | 1.940 | 0.480 | Peripheral (Peripherals) | Proteobacteria |
| OTU_188 | 5 | 6 | 1.581 | 0.480 | Peripheral (Peripherals) | Proteobacteria |
| OTU_13780 | 5 | 5 | -1.584 | 0.480 | Peripheral (Peripherals) | Proteobacteria |
| OTU_162 | 5 | 3 | -0.970 | 0.480 | Peripheral (Peripherals) | Actinobacteriota |
| OTU_95 | 31 | 5 | -0.013 | 0.499 | Peripheral (Peripherals) | Firmicutes |
| OTU_45 | 26 | 0 | 1.075 | 0.500 | Peripheral (Peripherals) | Firmicutes |
| OTU_18 | 6 | 5 | -1.464 | 0.500 | Peripheral (Peripherals) | Bacteroidota |
| OTU_37 | 6 | 5 | -1.584 | 0.500 | Peripheral (Peripherals) | Bacteroidota |
| OTU_137 | 4 | 0 | -1.193 | 0.500 | Peripheral (Peripherals) | Firmicutes |
| OTU_10081 | 4 | 6 | 0.000 | 0.500 | Peripheral (Peripherals) | Unknown |
| OTU_5335 | 33 | 1 | 0.197 | 0.501 | Peripheral (Peripherals) | Firmicutes |
| OTU_13331 | 36 | 1 | 0.329 | 0.526 | Peripheral (Peripherals) | Actinobacteriota |
| OTU_126 | 8 | 5 | -1.343 | 0.531 | Peripheral (Peripherals) | Proteobacteria |
| OTU_166 | 44 | 1 | 0.991 | 0.536 | Peripheral (Peripherals) | Firmicutes |
| OTU_85 | 14 | 0 | 0.250 | 0.541 | Peripheral (Peripherals) | Bacteroidota |
| OTU_4462 | 38 | 1 | 0.461 | 0.547 | Peripheral (Peripherals) | Firmicutes |
| OTU_17 | 35 | 1 | 0.064 | 0.549 | Peripheral (Peripherals) | Firmicutes |
| OTU_104 | 36 | 1 | 0.329 | 0.549 | Peripheral (Peripherals) | Firmicutes |
| OTU_39 | 46 | 1 | 1.123 | 0.552 | Peripheral (Peripherals) | Bacteroidota |
| OTU_151 | 38 | 0 | 0.869 | 0.554 | Peripheral (Peripherals) | Firmicutes |
| OTU_14797 | 36 | 0 | 1.075 | 0.557 | Peripheral (Peripherals) | Firmicutes |
| OTU_23 | 47 | 1 | 1.123 | 0.558 | Peripheral (Peripherals) | Firmicutes |
| OTU_4 | 5 | 0 | -0.987 | 0.560 | Peripheral (Peripherals) | Proteobacteria |
| OTU_19 | 39 | 0 | 1.075 | 0.560 | Peripheral (Peripherals) | Firmicutes |
| OTU_322 | 23 | 1 | -0.730 | 0.563 | Peripheral (Peripherals) | Actinobacteriota |
| OTU_11 | 42 | 1 | 0.726 | 0.568 | Peripheral (Peripherals) | Bacteroidota |
| OTU_10 | 42 | 1 | 0.726 | 0.568 | Peripheral (Peripherals) | Gracilibacteria |
| OTU_6595 | 30 | 0 | 1.694 | 0.571 | Peripheral (Peripherals) | Proteobacteria |
| OTU_310 | 7 | 6 | 0.000 | 0.571 | Peripheral (Peripherals) | Proteobacteria |
| OTU_147 | 36 | 1 | 0.064 | 0.579 | Peripheral (Peripherals) | Proteobacteria |
| OTU_7491 | 27 | 5 | -0.255 | 0.582 | Peripheral (Peripherals) | Proteobacteria |
| OTU_132 | 20 | 0 | 0.250 | 0.585 | Peripheral (Peripherals) | Bacteroidota |
| OTU_9289 | 26 | 0 | 0.457 | 0.589 | Peripheral (Peripherals) | Proteobacteria |
| OTU_74 | 45 | 0 | 2.106 | 0.593 | Peripheral (Peripherals) | Actinobacteriota |
| OTU_27 | 45 | 1 | 0.726 | 0.593 | Peripheral (Peripherals) | Actinobacteriota |
| OTU_124 | 48 | 1 | 0.858 | 0.602 | Peripheral (Peripherals) | Actinobacteriota |
| OTU_2 | 14 | 0 | -0.574 | 0.602 | Peripheral (Peripherals) | Proteobacteria |
| OTU_6 | 42 | 1 | 0.197 | 0.622 | Connector (Connectors) | Bacteroidota |
| OTU_158 | 52 | 1 | 0.858 | 0.624 | Connector (Connectors) | Firmicutes |
| OTU_98 | 33 | 5 | -0.496 | 0.628 | Connector (Connectors) | Firmicutes |
| OTU_50 | 30 | 5 | -0.255 | 0.638 | Connector (Connectors) | Firmicutes |
| OTU_8 | 15 | 0 | -0.368 | 0.640 | Connector (Connectors) | Cyanobacteria |
| OTU_60 | 48 | 5 | 0.350 | 0.643 | Connector (Connectors) | Proteobacteria |
| OTU_141 | 32 | 1 | -0.465 | 0.645 | Connector (Connectors) | Proteobacteria |
| OTU_414 | 35 | 0 | 0.869 | 0.647 | Connector (Connectors) | Firmicutes |
| OTU_78 | 34 | 0 | 1.075 | 0.652 | Connector (Connectors) | Proteobacteria |
| OTU_286 | 40 | 5 | -0.134 | 0.656 | Connector (Connectors) | Firmicutes |
| OTU_2718 | 42 | 0 | 0.250 | 0.659 | Connector (Connectors) | Proteobacteria |
| OTU_5 | 3 | 0 | -1.399 | 0.667 | Connector (Connectors) | Firmicutes |
| OTU_24 | 24 | 0 | 0.044 | 0.667 | Connector (Connectors) | Proteobacteria |

Note: Zi represents within-module connectivity, and Pi represents among-module connectivity. Nodes were classified into four distinct ecological roles: peripherals (Zi ≤ 2.5, Pi ≤ 0.62), connectors (Zi ≤ 2.5, Pi > 0.62), module hubs (Zi > 2.5, Pi ≤ 0.62), and network hubs (Zi > 2.5, Pi > 0.62).

Table S19. Nearest Sequenced Taxon Index (NSTI) scores for the 180 matched oral samples.

| Sample_ID | Panda_ID | Niche | Month | weighted_NSTI |
| --- | --- | --- | --- | --- |
| p1p04 | P1 | Plaque | 4 | 0.06399886 |
| p10p04 | P10 | Plaque | 4 | 0.06654878 |
| p11p04 | P11 | Plaque | 4 | 0.0709029 |
| p12p04 | P12 | Plaque | 4 | 0.05802291 |
| p2p04 | P2 | Plaque | 4 | 0.10084013 |
| p3p04 | P3 | Plaque | 4 | 0.06102143 |
| p4p04 | P4 | Plaque | 4 | 0.07637542 |
| p5p04 | P5 | Plaque | 4 | 0.01791494 |
| p6p04 | P6 | Plaque | 4 | 0.10810242 |
| p7p04 | P7 | Plaque | 4 | 0.0734767 |
| p8p04 | P8 | Plaque | 4 | 0.08022411 |
| p9p04 | P9 | Plaque | 4 | 0.06530747 |
| p1p05 | P1 | Plaque | 5 | 0.06640899 |
| p10p05 | P10 | Plaque | 5 | 0.01528041 |
| p11p05 | P11 | Plaque | 5 | 0.08821433 |
| p12p05 | P12 | Plaque | 5 | 0.08026765 |
| p2p05 | P2 | Plaque | 5 | 0.112475 |
| p3p05 | P3 | Plaque | 5 | 0.07883362 |
| p4p05 | P4 | Plaque | 5 | 0.07602359 |
| p5p05 | P5 | Plaque | 5 | 0.06685821 |
| p6p05 | P6 | Plaque | 5 | 0.08690482 |
| p7p05 | P7 | Plaque | 5 | 0.05016701 |
| p8p05 | P8 | Plaque | 5 | 0.07796607 |
| p9p05 | P9 | Plaque | 5 | 0.06385055 |
| p1p06 | P1 | Plaque | 6 | 0.06389001 |
| p10p06 | P10 | Plaque | 6 | 0.04497248 |
| p11p06 | P11 | Plaque | 6 | 0.04717736 |
| p12p06 | P12 | Plaque | 6 | 0.11030277 |
| p2p06 | P2 | Plaque | 6 | 0.08835961 |
| p3p06 | P3 | Plaque | 6 | 0.07267528 |
| p4p06 | P4 | Plaque | 6 | 0.07804853 |
| p5p06 | P5 | Plaque | 6 | 0.01907252 |
| p6p06 | P6 | Plaque | 6 | 0.0771825 |
| p7p06 | P7 | Plaque | 6 | 0.09074032 |
| p8p06 | P8 | Plaque | 6 | 0.06310973 |
| p9p06 | P9 | Plaque | 6 | 0.08090985 |
| p1p08 | P1 | Plaque | 8 | 0.07346468 |
| p10p08 | P10 | Plaque | 8 | 0.05347554 |
| p11p08 | P11 | Plaque | 8 | 0.06467501 |
| p12p08 | P12 | Plaque | 8 | 0.03845019 |
| p2p08 | P2 | Plaque | 8 | 0.06863862 |
| p3p08 | P3 | Plaque | 8 | 0.10702897 |
| p4p08 | P4 | Plaque | 8 | 0.05728562 |
| p5p08 | P5 | Plaque | 8 | 0.04477194 |
| p6p08 | P6 | Plaque | 8 | 0.04386021 |
| p7p08 | P7 | Plaque | 8 | 0.03434601 |
| p8p08 | P8 | Plaque | 8 | 0.07919186 |
| p9p08 | P9 | Plaque | 8 | 0.09372837 |
| p1p10 | P1 | Plaque | 10 | 0.09162818 |
| p10p10 | P10 | Plaque | 10 | 0.05907602 |
| p11p10 | P11 | Plaque | 10 | 0.06155096 |
| p12p10 | P12 | Plaque | 10 | 0.04685987 |
| p2p10 | P2 | Plaque | 10 | 0.04129347 |
| p3p10 | P3 | Plaque | 10 | 0.08593809 |
| p4p10 | P4 | Plaque | 10 | 0.10386883 |
| p5p10 | P5 | Plaque | 10 | 0.06592048 |
| p6p10 | P6 | Plaque | 10 | 0.05697706 |
| p7p10 | P7 | Plaque | 10 | 0.04241613 |
| p8p10 | P8 | Plaque | 10 | 0.04980412 |
| p9p10 | P9 | Plaque | 10 | 0.07547213 |
| p1p12 | P1 | Plaque | 12 | 0.0646021 |
| p10p12 | P10 | Plaque | 12 | 0.05538506 |
| p11p12 | P11 | Plaque | 12 | 0.05350047 |
| p12p12 | P12 | Plaque | 12 | 0.06265555 |
| p2p12 | P2 | Plaque | 12 | 0.05518223 |
| p3p12 | P3 | Plaque | 12 | 0.05768355 |
| p4p12 | P4 | Plaque | 12 | 0.04687778 |
| p5p12 | P5 | Plaque | 12 | 0.06247526 |
| p6p12 | P6 | Plaque | 12 | 0.05053643 |
| p7p12 | P7 | Plaque | 12 | 0.05173085 |
| p8p12 | P8 | Plaque | 12 | 0.05626164 |
| p9p12 | P9 | Plaque | 12 | 0.05056627 |
| p1s01 | P1 | Saliva | 1 | 0.07267638 |
| p10s01 | P10 | Saliva | 1 | 0.06583912 |
| p11s01 | P11 | Saliva | 1 | 0.02349383 |
| p12s01 | P12 | Saliva | 1 | 0.057553 |
| p2s01 | P2 | Saliva | 1 | 0.05739965 |
| p3s01 | P3 | Saliva | 1 | 0.04549542 |
| p4s01 | P4 | Saliva | 1 | 0.02008967 |
| p5s01 | P5 | Saliva | 1 | 0.03699978 |
| p6s01 | P6 | Saliva | 1 | 0.08096073 |
| p7s01 | P7 | Saliva | 1 | 0.04298094 |
| p8s01 | P8 | Saliva | 1 | 0.05762065 |
| p9s01 | P9 | Saliva | 1 | 0.05368696 |
| p1s02 | P1 | Saliva | 2 | 0.09040469 |
| p10s02 | P10 | Saliva | 2 | 0.08048956 |
| p11s02 | P11 | Saliva | 2 | 0.04986894 |
| p12s02 | P12 | Saliva | 2 | 0.05267552 |
| p2s02 | P2 | Saliva | 2 | 0.06565064 |
| p3s02 | P3 | Saliva | 2 | 0.05134984 |
| p4s02 | P4 | Saliva | 2 | 0.07620779 |
| p5s02 | P5 | Saliva | 2 | 0.04642183 |
| p6s02 | P6 | Saliva | 2 | 0.0513085 |
| p7s02 | P7 | Saliva | 2 | 0.04840567 |
| p8s02 | P8 | Saliva | 2 | 0.07174653 |
| p9s02 | P9 | Saliva | 2 | 0.04160792 |
| p1s03 | P1 | Saliva | 3 | 0.07034583 |
| p10s03 | P10 | Saliva | 3 | 0.08362212 |
| p11s03 | P11 | Saliva | 3 | 0.07055825 |
| p12s03 | P12 | Saliva | 3 | 0.05254801 |
| p2s03 | P2 | Saliva | 3 | 0.09624637 |
| p3s03 | P3 | Saliva | 3 | 0.06156232 |
| p4s03 | P4 | Saliva | 3 | 0.06265255 |
| p5s03 | P5 | Saliva | 3 | 0.06457698 |
| p6s03 | P6 | Saliva | 3 | 0.07624963 |
| p7s03 | P7 | Saliva | 3 | 0.06968114 |
| p8s03 | P8 | Saliva | 3 | 0.07759633 |
| p9s03 | P9 | Saliva | 3 | 0.05144204 |
| p1s04 | P1 | Saliva | 4 | 0.08903775 |
| p10s04 | P10 | Saliva | 4 | 0.08306477 |
| p11s04 | P11 | Saliva | 4 | 0.07458232 |
| p12s04 | P12 | Saliva | 4 | 0.05530448 |
| p2s04 | P2 | Saliva | 4 | 0.08974301 |
| p3s04 | P3 | Saliva | 4 | 0.07132408 |
| p4s04 | P4 | Saliva | 4 | 0.06898165 |
| p5s04 | P5 | Saliva | 4 | 0.06838762 |
| p6s04 | P6 | Saliva | 4 | 0.07776114 |
| p7s04 | P7 | Saliva | 4 | 0.06716301 |
| p8s04 | P8 | Saliva | 4 | 0.09230269 |
| p9s04 | P9 | Saliva | 4 | 0.06235946 |
| p1s05 | P1 | Saliva | 5 | 0.09063191 |
| p10s05 | P10 | Saliva | 5 | 0.06165092 |
| p11s05 | P11 | Saliva | 5 | 0.05775065 |
| p12s05 | P12 | Saliva | 5 | 0.13329923 |
| p2s05 | P2 | Saliva | 5 | 0.1043887 |
| p3s05 | P3 | Saliva | 5 | 0.0797179 |
| p4s05 | P4 | Saliva | 5 | 0.13132456 |
| p5s05 | P5 | Saliva | 5 | 0.08072474 |
| p6s05 | P6 | Saliva | 5 | 0.07474545 |
| p7s05 | P7 | Saliva | 5 | 0.06922483 |
| p8s05 | P8 | Saliva | 5 | 0.06545724 |
| p9s05 | P9 | Saliva | 5 | 0.06456382 |
| p1s06 | P1 | Saliva | 6 | 0.06955306 |
| p10s06 | P10 | Saliva | 6 | 0.04443773 |
| p11s06 | P11 | Saliva | 6 | 0.04842673 |
| p12s06 | P12 | Saliva | 6 | 0.05366883 |
| p2s06 | P2 | Saliva | 6 | 0.08402666 |
| p3s06 | P3 | Saliva | 6 | 0.06468507 |
| p4s06 | P4 | Saliva | 6 | 0.11143227 |
| p5s06 | P5 | Saliva | 6 | 0.01876435 |
| p6s06 | P6 | Saliva | 6 | 0.03465084 |
| p7s06 | P7 | Saliva | 6 | 0.09201899 |
| p8s06 | P8 | Saliva | 6 | 0.08853505 |
| p9s06 | P9 | Saliva | 6 | 0.0790188 |
| p1s08 | P1 | Saliva | 8 | 0.08298894 |
| p10s08 | P10 | Saliva | 8 | 0.05192944 |
| p11s08 | P11 | Saliva | 8 | 0.0558535 |
| p12s08 | P12 | Saliva | 8 | 0.03928472 |
| p2s08 | P2 | Saliva | 8 | 0.07515259 |
| p3s08 | P3 | Saliva | 8 | 0.11682877 |
| p4s08 | P4 | Saliva | 8 | 0.10006272 |
| p5s08 | P5 | Saliva | 8 | 0.0530217 |
| p6s08 | P6 | Saliva | 8 | 0.04702497 |
| p7s08 | P7 | Saliva | 8 | 0.03632595 |
| p8s08 | P8 | Saliva | 8 | 0.05483887 |
| p9s08 | P9 | Saliva | 8 | 0.08729434 |
| p1s10 | P1 | Saliva | 10 | 0.05942176 |
| p10s10 | P10 | Saliva | 10 | 0.05866819 |
| p11s10 | P11 | Saliva | 10 | 0.05466691 |
| p12s10 | P12 | Saliva | 10 | 0.0481295 |
| p2s10 | P2 | Saliva | 10 | 0.07309835 |
| p3s10 | P3 | Saliva | 10 | 0.07021395 |
| p4s10 | P4 | Saliva | 10 | 0.08497205 |
| p5s10 | P5 | Saliva | 10 | 0.06649821 |
| p6s10 | P6 | Saliva | 10 | 0.05086249 |
| p7s10 | P7 | Saliva | 10 | 0.0471616 |
| p8s10 | P8 | Saliva | 10 | 0.05542324 |
| p9s10 | P9 | Saliva | 10 | 0.04038539 |
| p1s12 | P1 | Saliva | 12 | 0.05991234 |
| p10s12 | P10 | Saliva | 12 | 0.05398769 |
| p11s12 | P11 | Saliva | 12 | 0.03309914 |
| p12s12 | P12 | Saliva | 12 | 0.06288141 |
| p2s12 | P2 | Saliva | 12 | 0.05874939 |
| p3s12 | P3 | Saliva | 12 | 0.0498928 |
| p4s12 | P4 | Saliva | 12 | 0.05523344 |
| p5s12 | P5 | Saliva | 12 | 0.05856038 |
| p6s12 | P6 | Saliva | 12 | 0.05599355 |
| p7s12 | P7 | Saliva | 12 | 0.05427327 |
| p8s12 | P8 | Saliva | 12 | 0.05558094 |
| p9s12 | P9 | Saliva | 12 | 0.05464962 |
